# Isolation and sequencing of Dashli virus, a novel Sicilian-like virus in sandflies from Iran; genetic and phylogenetic evidence for the creation of one novel species within the *Phlebovirus* genus in the *Phenuiviridae* family

**DOI:** 10.1371/journal.pntd.0005978

**Published:** 2017-12-27

**Authors:** Cigdem Alkan, Vahideh Moin Vaziri, Nazli Ayhan, Mehdi Badakhshan, Laurence Bichaud, Nourina Rahbarian, Ezat-Aldin Javadian, Bulent Alten, Xavier de Lamballerie, Remi N. Charrel

**Affiliations:** 1 UMR "Unité des Virus Emergents" (UVE Aix-Marseille Univ—IRD 190—Inserm 1207—EHESP), Marseille, France; 2 Fondation IHU Mediterranee Infection, APHM Public Hospitals of Marseille, Marseille, France; 3 Department of Parasitology and Mycology, School of Medicine, Shahid Beheshti University of Medical Sciences, Tehran, Iran; 4 Department of Medical Entomology and Vector Control, School of Public Health and Institute of Public Health Research, Tehran University of Medical Sciences, Tehran, Iran; 5 Cellular and Molecular Biology Research Center, Shahid Beheshti University of Medical Sciences, Tehran, Iran; 6 Faculty of Science, Department of Biology, Ecology Section, ESR Laboratories, Hacettepe University, Ankara, Turkey; University of Notre Dame, UNITED STATES

## Abstract

Phlebotomine sandflies are vectors of phleboviruses that cause sandfly fever or meningitis with significant implications for public health. Although several strains of these viruses had been isolated in Iran in the late 1970's, there was no recent data about the present situation at the outset of this study. Entomological investigations performed in 2009 and 2011 in Iran collected 4,770 sandflies from 10 different regions. Based on morphological identification, they were sorted into 315 pools according to species, sex, trapping station and date of capture. A phlebovirus, provisionally named Dashli virus (DASHV), was isolated from one pool of *Sergentomyia spp*, and subsequently DASHV RNA was detected in a second pool of *Phlebotomus papatasi*. Genetic and phylogenetic analyses based on complete coding genomic sequences indicated that (i) DASHV is most closely related to the Iranian isolates of Sandfly fever Sicilian virus [SFSV], (ii) there is a common ancestor to DASHV, Sandfly fever Sicilian- (SFS) and SFS-like viruses isolated in Italy, India, Turkey, and Cyprus (lineage I), (iii) DASHV is more distantly related with Corfou and Toros viruses (lineage II) although common ancestry is supported with 100% bootstrap, (iii) lineage I can be subdivided into sublineage Ia including all SFSV, SFCV and SFTV except those isolated in Iran which forms sublineage Ib (DASHV). Accordingly, we suggest to approve *Sandfly fever Sicilian virus* species consisting of the all aforementioned viruses. Owing that most of these viruses have been identified in human patients with febrile illness, DASHV should be considered as a potential human pathogen in Iran.

## Author summary

Phlebotomine sandflies are vectors of phleboviruses that cause sandfly fever or meningitis with significant implications for public health. Although several strains of these viruses had been isolated in Iran in the late 1970's, there was no recent data about the present situation at the outset of this study. Entomological investigations performed in 2009 and 2011 in Iran collected 4,770 sandflies from 10 different regions. A phlebovirus, provisionally named Dashli virus (DASHV), was isolated / detected in two pools. DASHV strain was isolated in cell culture and complete genome sequence was determined. Sequence analysis indicated that (i) DASHV is most closely related to the Iranian isolates of Sandfly fever Sicilian virus [SFSV], a virus that is known to cause self-resolutive incapacitating febrile illness in humans, (ii) there is a common ancestor to DASHV and all other variants of SFSV isolated in Italy, India, Turkey, and Cyprus (lineage I), (iii) DASHV is more distantly related with Corfou and Toros viruses (lineage II) although common ancestry is supported with 100% bootstrap, (iii) lineage I can be subdivided into sublineage Ia including all SFSV strains, whereas Iranian viruses are most closely related and should be individualized as DASHV (sublineage Ib). Although discovered first in the 1940's, SFSV is still listed as "tentative species" by the International Committee for Taxonomy of Viruses. Based on the results described in this study, we propose to approve *Sandfly fever Sicilian virus* species. Owing that most of these viruses have been identified in human patients with febrile illness, DASHV should be considered as a potential human pathogen in Iran.

## Introduction

The genus *Phlebovirus*, family *Phenuiviridae* currently contains 9 recognised virus species (grouping 37 viruses), and 33 tentative species [1]. At least 9 new viruses (Adana, Alcube, Arrabida, Fermo, Granada, Medjerda Valley, Punique, Toros, Zerdali) have also been isolated and appear to belong to the three groups primarily transmitted by phlebotomines in the Old World [2–9].

In the Old World, sandfly-borne phleboviruses are distributed in the Mediterranean region, Africa, the Indian subcontinent, the Middle East and central Asia, where they are transmitted by sandflies belonging to the genera *Phlebotomus and Sergentomyia*. In contrast, New World sandfly-borne phleboviruses are transmitted by phlebotomines of the genus *Lutzomyia*. There is a strict discrimination between Old World and New World phleboviruses due to the exclusive distribution of their respective vectors. Current, phleboviruses pathogenic for humans include: (i) Alenquer (ALEV), Candiru (CDUV), Escharte (ESCV), Serra Norte (SRNV), Morumbi (MRBV), Maldonado (MLOV), Chagres (CHGV), Adria (ADRV), Naples (SFNV), Sicilian (SFSV) and Toscana viruses (TOSV), all of which are transmitted by sandflies, (ii) Punta Toro (PTV) and Rift Valley fever (RVFV) viruses which are transmitted by mosquitoes, (iii) Bhanja (BHAV), Severe fever thrombocytopenia syndrome (SFTSV), and Heartland (HRTV) viruses which are transmitted by ticks [10–15]

Amongst the Old World sandfly-borne phleboviruses, two species (*Sandfly fever Naples* and *Salehabad*) and two tentative species (Sandfly fever Sicilian [SFSV] and Corfou [CFUV]) are listed in the IX^th^ report of the International Classification for Taxonomy of Viruses (ICTV) [1]. SFSV and SFNV are associated with self-limiting febrile illness, whereas Toscana virus (TOSV) is often associated with neurological manifestations either central or peripheral [16]; TOSV is the most prevalent sandfly-borne arbovirus in Europe, particularly in countries bordering the Mediterranean [17]. Although SFNV, discovered in 1942 during World War II (WWII), was for a long time considered to be a prominent cause of incapacitating fever in the Mediterranean region, the last reported case was confirmed in 1990 [18]. It is not impossible that SFNV would have gone extinct since. In contrast, SFSV remains endemic in the Mediterranean basin, the Middle East, Central Asia and Europe [17]. SFSV was first isolated from the sera of a sick US soldier in Egypt in 1943 during WWII, and later was isolated again in Sicily during an outbreak of febrile illness among US-army troops [19]. There is accumulating direct (virus isolation or molecular detection) and indirect (seroprevalence studies) evidence that viruses closely related to but clearly distinct from SFSV are widespread in the Mediterranean region and the Middle East. Outbreaks and sporadic human cases occurred in Cyprus (Sandfly fever Cyprus virus [SFCV]), in Turkey (Sandfly fever Turkey virus [SFTV]), in Ethiopia (SFSV-Ethiopia) [20–23]. Corfou virus [CFUV], discovered in 1985 in *Phlebotomus neglectus* on the eponymous Greek island, was never associated with human infection [24]; however, viral RNA was detected in the CSF of a patient and the corresponding virus was provisionally named Chios-A virus; sequences of CFUV and Chios-A virus were very similar [3, 25]. It is also important to underline that neutralisation test can easily distinguish SFSV from CFUV, despite less discriminative serological methods (ELISA, HI, IIF, CF) cannot [24].

Several pheboviruses have been isolated from sandflies in Iran: SALV, KARV, and THEV in 1959, and SFSV in 1975 [26]. Active circulation of these viruses and SFNV was also supported by finding neutralizing antibodies in human sera [27, 28]. To investigate whether these viruses or new ones are currently circulating in sandflies in Iran, field campaigns were organized in different localities during the summers of 2009 and 2011. This article presents the molecular detection, virus isolation, complete genome sequencing and subsequent genetic and phylogenetic analysis of a novel SFSV-like virus, provisionally named Dashli virus (DASHV) from the village of Dashliborun where infected sandflies were collected.

## Results

### Sandfly trapping and virus detection

**Fig 1:**
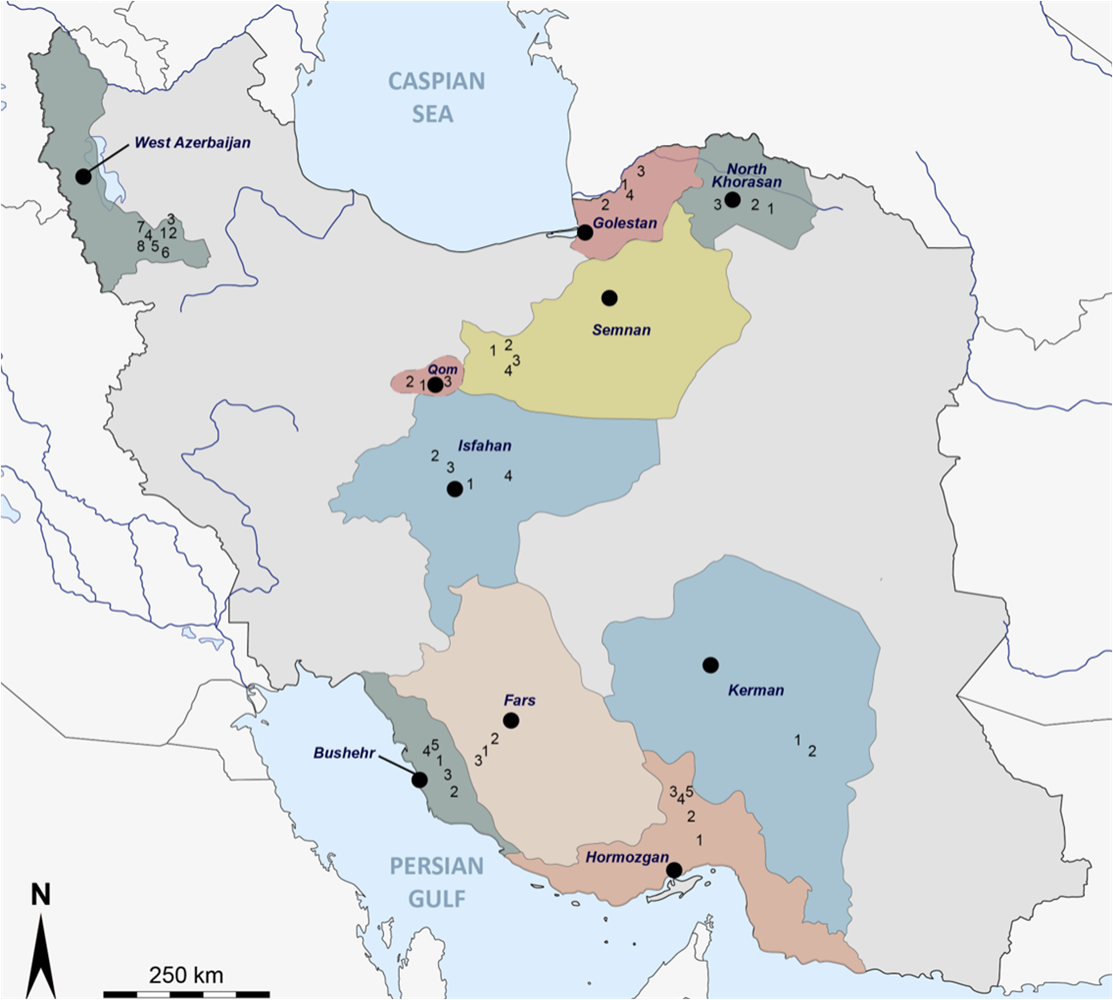
Geographic representation of the sandfly collection regions in Iran.

A total of 4,770 (3,158 females and 1,162 males) sandflies were collected and identified morphologically. They were allocated to 315 pools (198 female and 117 male pools). The number of sandflies and pools originating from individual villages are shown in Table 1 and Fig 1. The most abundant species were *P*. *papatasi* (57.57%) and *Sergentomyia* spp. (31.05%). The less abundant species were; *P*. *alexandri*, *P*. *mascitti*, *P*. *tobbi*, *P*. *mongolensis*, *P*. *sergenti*, *P*. *caucasicus*, *P*. *major*, *P*. *bergeroti*, and *P*. *kandelakii*. Pool #131 that consisted of 30 non-engorged female *Sergentomyia spp*. trapped in the Shordakesh in Dasliboroun village, in July 2011 was positive with primers pair N-Phlebo1S/ 1R [29]. The resulting 502-nt sequence in the polymerase gene was most closely related to Sandfly fever Turkey virus (SFTV—GenBank acc no: GQ847513) with sequence identities of 94% and 81% at the AA and nt levels, respectively. Using the rt-RT-PCR assay designed for the specific detection of DASHV, pool #131 was confirmed as positive and pool#94 was also found positive (Ct values < 28). Four-fold dilutions of the total nucleic acids derived from these 2 pools were tested using DASHV rt-RT-PCR, and dilutions up to 1:1,024 were positive (1:4,096 dilution was negative) with Ct values ranging from 25.78 to 34.65 (S1 Fig). Pool # 94 consisted of 30 non-engorged females of *P*. *papatasi* which were also collected at the same location as pool #131 on the same day (06 July 2011). The rt-RT-PCR positive product of pool#94 was sequenced and the obtained 128 nucleotides were 100% identical with the homologous sequence corresponding to pool#131.

Assuming that only one sandfly was infected in each of the pools #94 and #131, the global sandfly infection rate for the DASHV in this study was estimated to be 0.04%. When considering only *Phlebotomus spp* (not *Sergentomyia spp*) in the Golestan region, the infection rate raised to 0.22% (2/904).

**Table 1:** Distribution of sandfly specimens and pools according to the sampling locations in Iran.

				Number of sandflies		Number of pools	
Province	City	Village	Species	Female	Male	Female	Male
Qum	Qum	Sub urban	*P*. *papatasi*	4	-	1	-
Qum	Qum	Jannat abad	*P*. *papatasi*	30	60	1	2
			*Sergentomiya spp*.	41	7	2	1
Qum	Qum	Koohe sefid	*P*. *papatasi*	103	50	5	2
			*Sergentomiya spp*.	103	30	3	1
			*P*. *alexandri*	10	-	1	-
			*P*. *masciti*	1	-	1	-
Semnan	Garmsar	Rikan	*P*. *papatasi*	60	74	2	3
			*Sergentomiya spp*.	6	30	1	1
			*P*. *tobbi*	-	5	-	1
			*P*. *sergenti*	1	-	1	-
Semnan	Garmsar	Emamzade abdollah	*P*. *papatasi*	221	180	8	6
			*Sergentomiya spp*.	69	36	3	1
			*P*. *alexandri*	2	-	1	-
			*Unknown*	1	-	1	-
Semnan	Garmsar	Shah sefid	*P*. *papatasi*	90	60	3	2
			*Sergentomiya spp*.	43	13	2	1
			*P*. *alexandri*	1	-	1	-
			*P*. *sergenti*	1	-	1	-
Golestan	Dasliboroun	Dasliboroun	*P*. *papatasi*	26	19	1	1
			*Sergentomiya spp*.	97	6	4	1
Golestan	Dasliboroun	Daneshmand	*P*. *papatasi*	47	22	2	1
			*Sergentomiya spp*.	52	6	2	1
Golestan	Dasliboroun	Kheir khaje	*P*. *papatasi*	10	5	1	1
			*Sergentomiya spp*.	2	-	1	-
			*P*. *caucasicus*	-	1	-	1
Golestan	Dasliboroun	Shordakesh	*P*. *papatasi*	440	328	16	11
			*Sergentomiya spp*.	1093	196	37	7
			*P*. *mongolensis*	-	5	-	1
			*Unknown*	29	-	1	-
			*P*. *caucasicus*	5	-	1	-
			*P*. *sergenti*	-	1	-	1
North Khorasan	Esfarayen	Zotg abad	*P*. *papatasi*	15	22	1	1
			*Sergentomiya spp*.	11	2	1	1
			*P*. *caucasicus*	-	1	-	1
North Khorasan	Esfarayen	Charborj	*P*. *papatasi*	22	-	1	-
			*Sergentomiya spp*.	6	30	1	1
Bushehr	Borazjan	Zirah	*P*.*bergeroti*	-	1	-	1
			*P*.*papatasi*	-	1	-	1
			*Sergentomiya spp*	4	-	1	-
Bushehr	Borazjan	Dehrood	*Sergentomiya spp*	2	2	1	1
			*P*.*papatasi*	7	-	1	-
Bushehr	Borazjan	Booshkan	*Sergentomiya spp*.	5	8	2	1
			*P*.*mongolensis*	-	11	-	2
			*P*.*alexandri*	6	-	2	-
			*P*.*papatasi*	31	9	2	2
			*P*.*sergenti*	1	-	1	-
			*Unknown paraphlebotomus*	9	-	1	-
Bushehr	Borazjan	Nanizak	*P*.*mongolensis*	-	3	-	1
			*P*.*caucasicus s*. *l*.	-	3	-	1
			*P*.*papatasi*	1	-	1	-
			*P*.*alexandri*	1	-	1	-
			*Sergentomiya spp*.	19	2	1	2
Bushehr	Borazjan	Arghoon	*P*.*alexandri*	2	-	1	-
			*Sergentomiya spp*.	1	-	1	-
Esfahan	Koohpaye	Jabal	*P*.*sergenti*	4	3	1	1
			*P*.*caucasicus s*.*l*.	3	-	1	-
			*P*.*papatasi*	7	-	1	-
			*P*.*major s*.*l*.	1	-	1	-
Esfahan	Gazborkhar	Sin	*P*.*papatasi*	-	4	-	1
			*P*.*sergenti*	1	-	1	-
Esfahan	Esfahan	Suburban	*P*.*papatasi*	26	1	1	1
			*Sergentomiya spp*.	9	2	1	1
			*P*. *mongolensis*	-	1	-	1
			*P*.*alexandri*	7	-	1	-
			*P*.*bergeroti*	-	5	-	1
Hormozgan	Hajiabad	Nesa	*Sergentomiya spp*	26	17	1	1
			*P*.*alexandri*	36	-	2	-
			*P*.*mongolensis*	-	9	-	1
			*P*.*bergeroti*	15	11	1	1
			*P*.*papatasi*	47	17	2	1
			*P*.*caucasicus s*.*l*.	1	-	1	-
			*P*.*sergenti*	30	-	1	-
			*Unknown*	1	-	1	-
Hormozgan	Hajiabad	Shahrood	*P*.*caucasicus s*.*l*	1	-	1	-
			*Sergentomiya spp*	8	9	1	1
			*P*.*mongolensis*	-	3	-	1
			*P*.*alexandri*	30	-	1	-
			*P*.*papatasi*	2	11	1	1
			*P*.*sergenti*	-	20	-	1
			*P*.*bergeroti*	-	1	-	1
Hormozgan	Hajiabad	Tashquieh	*P*.*papatasi*	111	45	4	3
			*P*.*sergenti*	1	3	1	1
			*P*.*alexandri*	4	-	2	-
			*P*.*mongolensis*	-	2	-	1
			*Unknown paraphlebotomus*	1	-	1	-
Hormozgan	Hajiabad	Jaein	*P*.*papatasi*	62	50	3	2
			*P*.*alexandri*	1	-	1	-
			*Sergentomiya spp*	5	1	1	1
			*P*.*caucasicus s*.*l*	1	-	1	-
			*Unknown*	1	-	1	-
Hormozgan	Bandar-abbas	Chooj	*Sergentomiya spp*	1	-	1	-
Fars	Farash-band	Shoor-abad	*P*.*papatasi*	26	27	1	1
			*Sergentomiya spp*.	2	-	1	-
			*P*.*alexandri*	1	-	1	-
Fars	Farash band	Jaein	*P*.*papatasi*	30	-	1	-
Fars	Farash band	-	*Sergentomiya spp*	6	7	1	1
		-	*P*.*papatasi*	-	14	-	1
		-	*P*.*sergenti*	1	-	1	-
Fars	Farash-band	EmamZadeh	*P*.*papatasi*	6	2	1	1
			*P*.*caucasicus s*. *l*.	1	-	1	-
			*Sergentomiya spp*	3	1	1	1
			*P*.*alexandri*	11	2	1	1
			*P*.*sergenti*	-	2	-	1
Western Azerbaijan	Miandoab	Sarchenar	*P*.*papatasi*	68	55	5	8
Western Azerbaijan	Miandoab	Mirza-nezam	*P*.*papatasi*	2	9	2	3
			*P*.*major s*.*l*.	3	-	2	-
			*Sergentomiya spp*.	4	-	2	-
			*P*.*caucasicus s*.*l*	4	-	1	-
			*P*.*sergenti*	4	-	1	-
			*P*. *kandelakii*	1	-	1	-
Western Azerbaijan	Miandoab	Hamidloo	*P*.*papatasi*	6	7	1	2
			*P*.*sergenti*	6	-	1	-
Western Azerbaijan	Miandoab	Badam	*P*.*papatasi*	9	1	1	1
Western Azerbaijan	Miandoab	Nuwroozloo	*P*.*papatasi*	7	3	1	1
Western Azerbaijan	Miandoab	Esmailkandi	*P*.*papatasi*	11	15	2	2
Western Azerbaijan	Miandoab	Ghopi-Babaali	*P*.*papatasi*	11	6	1	2
Western Azerbaijan	Miandoab	Zeynalkandi	*P*.*papatasi*	4	-	1	-
Kerman	Bam	Bravat	*Sergentomiya spp*	-	2	-	1
			*P*.*papatasi*	14	4	1	1
			*P*.*sergenti*	-	2	-	1
Kerman	Bam	Suburb	*Sergentomiya spp*	6	-	1	-
			*P*.*papatasi*	9	7	1	1
			*P*.*sergenti*	3	2	1	1
			*P*.*caucasicus s*.*l*	3	-	1	-
**Total**				**3158**	**1612**	**198**	**117**

### Virus isolation

The 12.5 cm^2^ flask of Vero cells inoculated directly with pool #131 showed a clear cytopathic effect (CPE) at day 4 after inoculation. The supernatant was used to seed one passage into 12.5 cm^2^ flask and this was done again until passage 4. Freeze-dried vials of infectious supernatant medium were prepared and included in the collection of the European Virus Archive (www.european-virus-archive.com/) where they are publicly available for academic research. Pool#94 was also inoculated onto Vero cells but neither CPE nor viral RNA could be detected after 4 consecutive passages.

### Complete genome sequencing

The complete genomic sequence of DASHV consisted of 6,444nts, 4,413 nts and 1,802 nts for the L, M and S segments, respectively (GenBank acc. no KP771821, KP771822, and KP771823). The polymerase gene encoded a 6,270-nt long ORF (2,090 AA), whereas the glycoprotein gene encoded a 1,342-nt long ORF (4,026 AA, further cleaved into a 531-AA long Gn and a 478-AA long Gc). The small segment encoded a 738-nt and an 801-nt long ORF which were translated to nucleocapsid protein (246 AA) and nonstructural protein 267 AA), respectively.

### Genetic distances

Pairwise distances of the nt- and AA-sequences are presented in S1 Table. Amino acid distances between DASHV compared with SFSV, SFTV and SFCV were ≤19,8% (L), ≤39,7% (Gn), ≤31,6% (Gc), ≤15,0% (N), and ≤36,2% (NS). Amino acid distances between DASHV and other phleboviruses were much higher: ≥43,2% (L), ≥61,5% (Gn), ≥45,4% (Gc) and, ≥ 46,9% (N), ≥73,5% (NS).

Gene by gene comparative distance analysis showed that pairwise distances of DASHV *vs* SFSV and SFS-like viruses were consistently lower than the lowest distances observed between DASHV and phleboviruses other than SFSV / SFS-like viruses. The distances between DASHV and SFS-like viruses from outside Iran were between 4.5–19.8% and 18.3–29.6% for the AA and nt sequences of the L, M, and S proteins (S1 Table). However these distances dropped to 0.0% and 9.3% for the N protein and 4.2% and 12.6% for the Ns protein, for AA and nt, respectively. In addition, the lowest interspecific distances; 40.0% (L), 46.2% (Gn), 33.6% (Gc), 35.8% (N), and 54.8% (Ns) among ICTV-recognized phlebovirus species [2] were higher than the lowest distances observed between DASHV and SFSV / SFS-like viruses. In addition, the lowest interspecific distances; 40.0% (L), 46.2% (Gn), 33.6% (Gc), 35.8% (N), and 54.8% (Ns) among ICTV-recognized phleboviruses [2] were higher than the highest distances observed between DASHV and SFSV / SFS-like viruses.

### Phylogenetic analysis

ICTV recognized species are clearly segregated in the phylogenies where they are supported by high bootstraps (100%) except for RVFV; thus our results are congruent with the previously reported topologies [14, 15, 30, 31].

**Fig 2:**
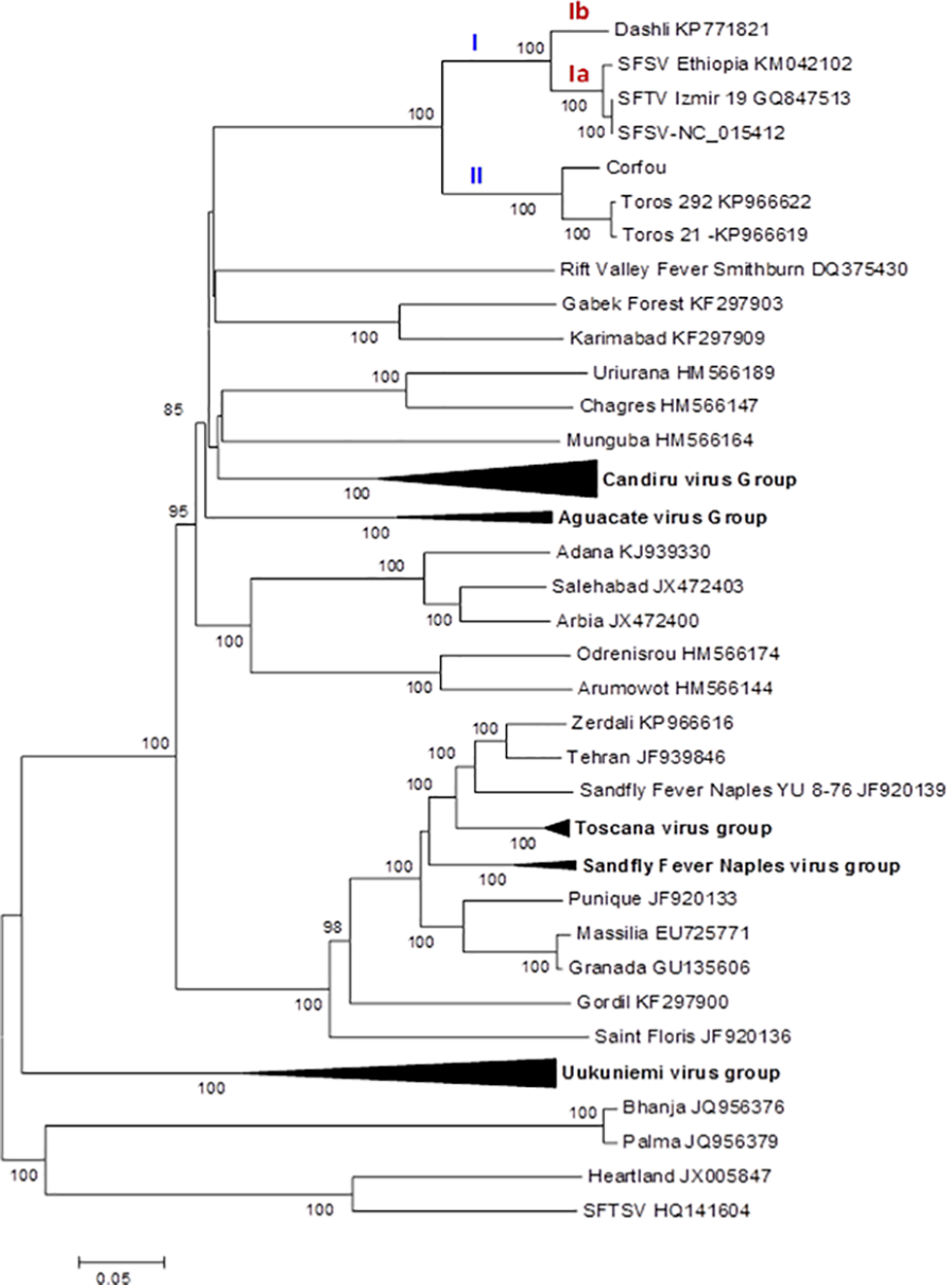
Phylogenetic analysis of the phlebovirus amino acid sequences: L protein.

**Fig 3:**
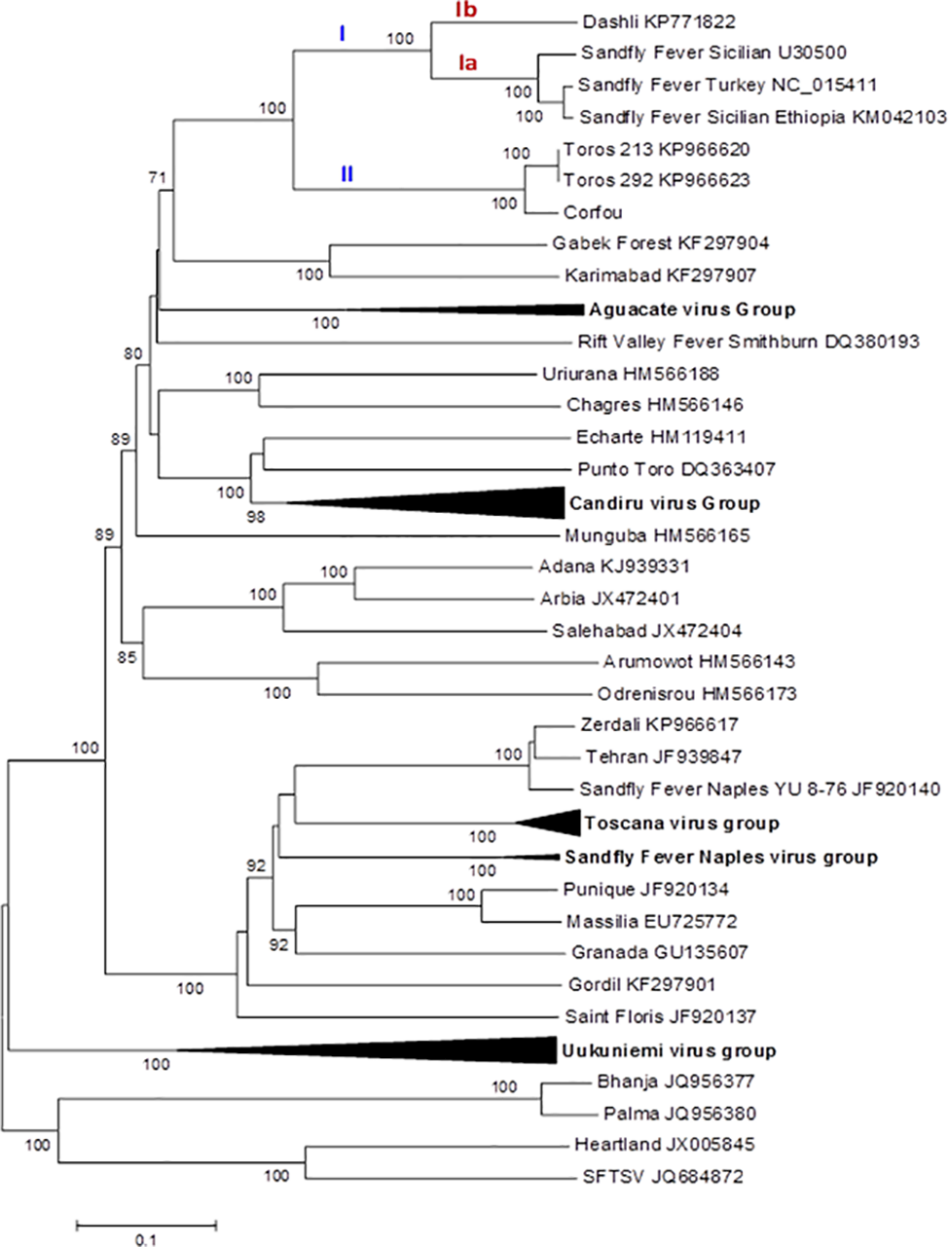
Phylogenetic analysis of the phlebovirus amino acid sequences: Gn protein.

**Fig 4:**
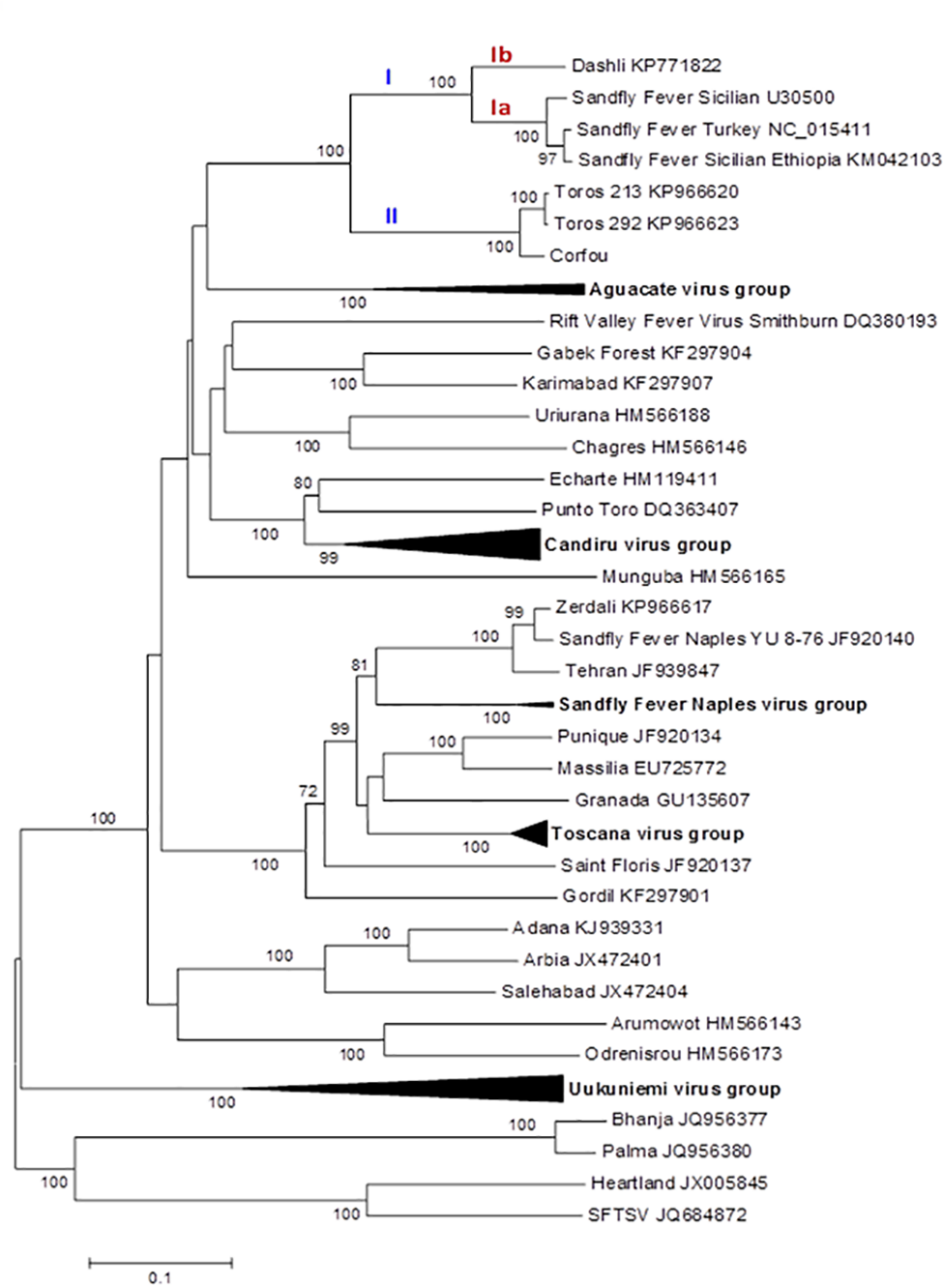
Phylogenetic analysis of the phlebovirus amino acid sequences: Gc protein.

**Fig 5:**
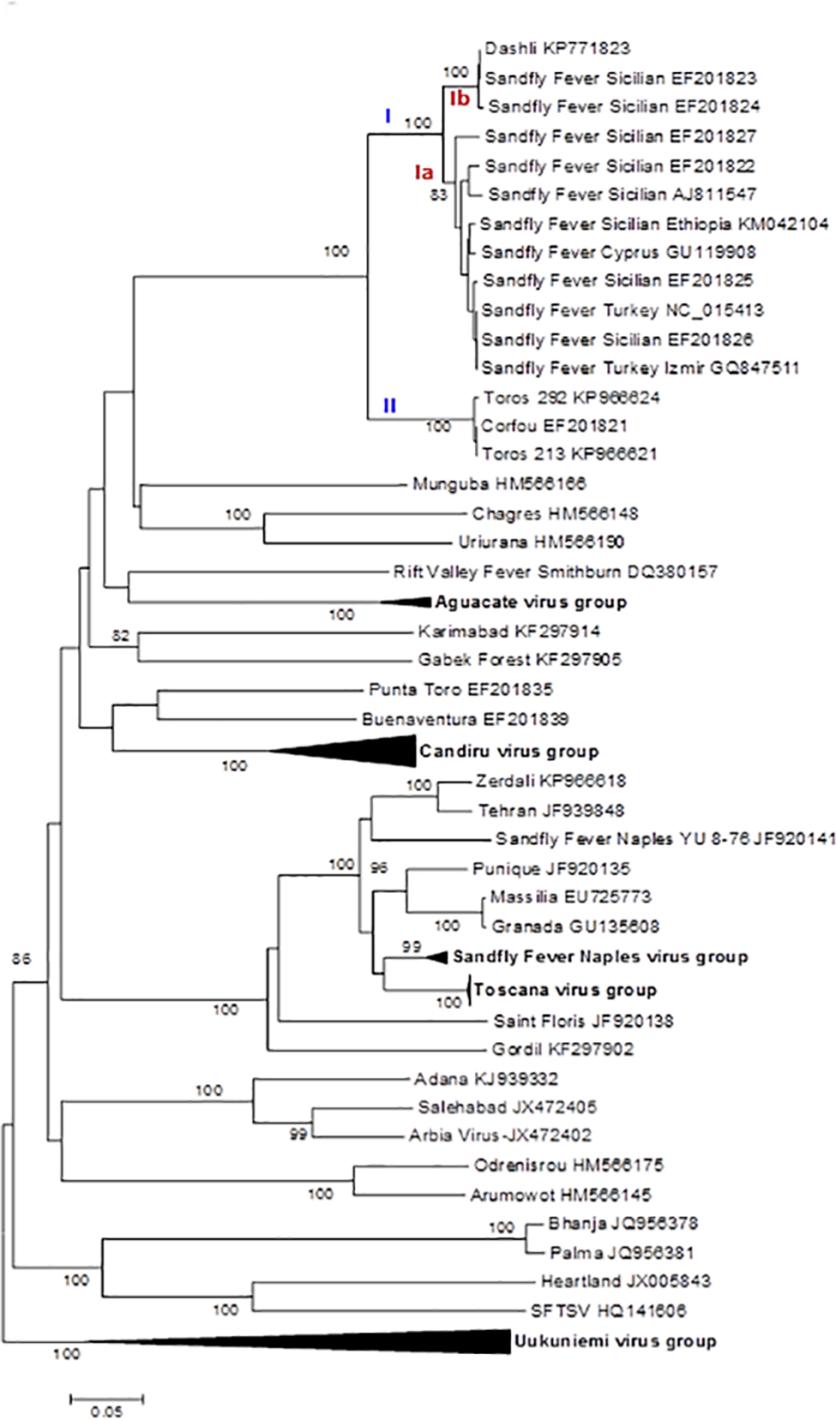
Phylogenetic analysis of the phlebovirus amino acid sequences: Nucleocapsid protein.

**Fig 6:**
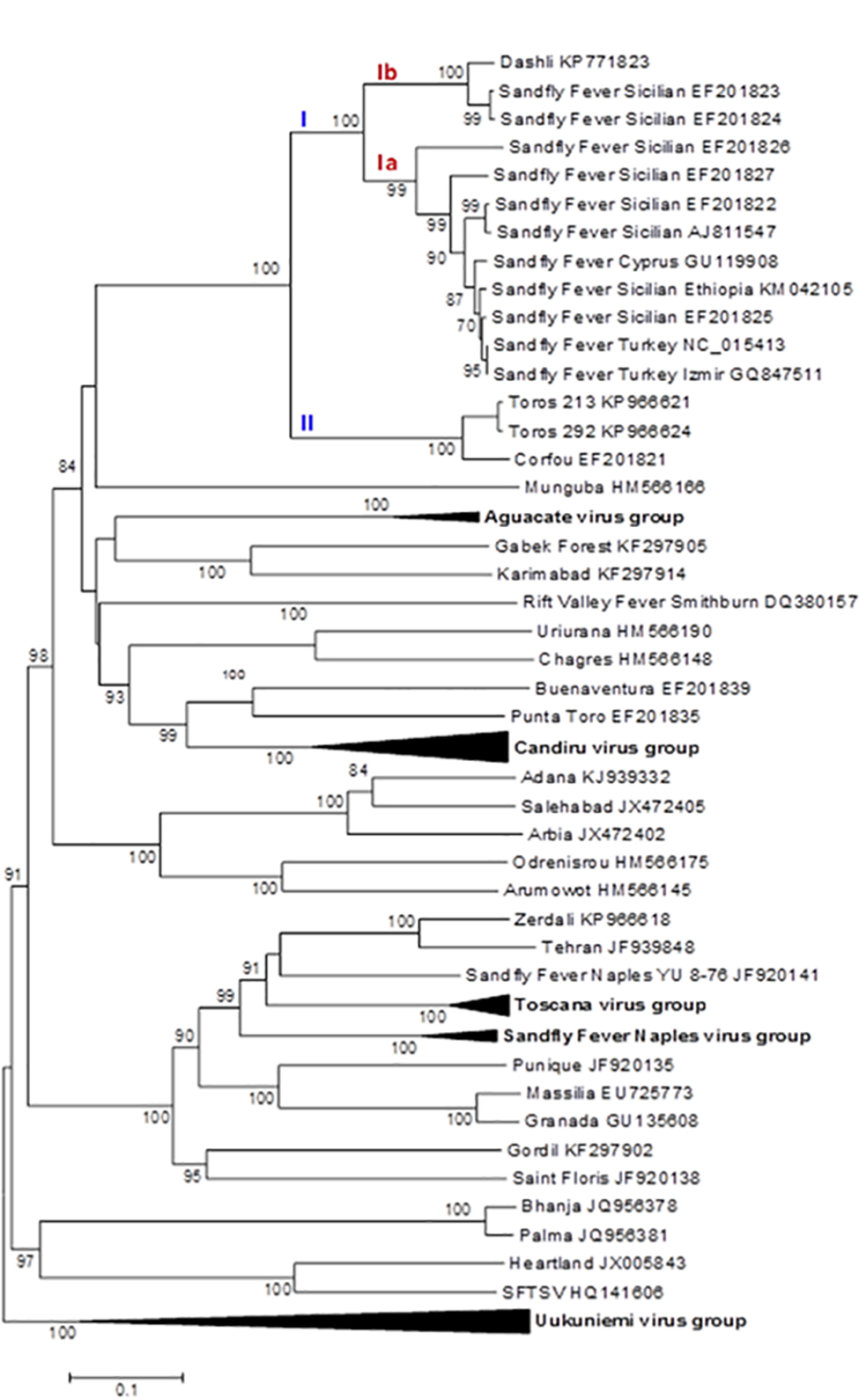
Phylogenetic analysis of the phlebovirus amino acid sequences: Non-structural protein.

**Fig 7:**
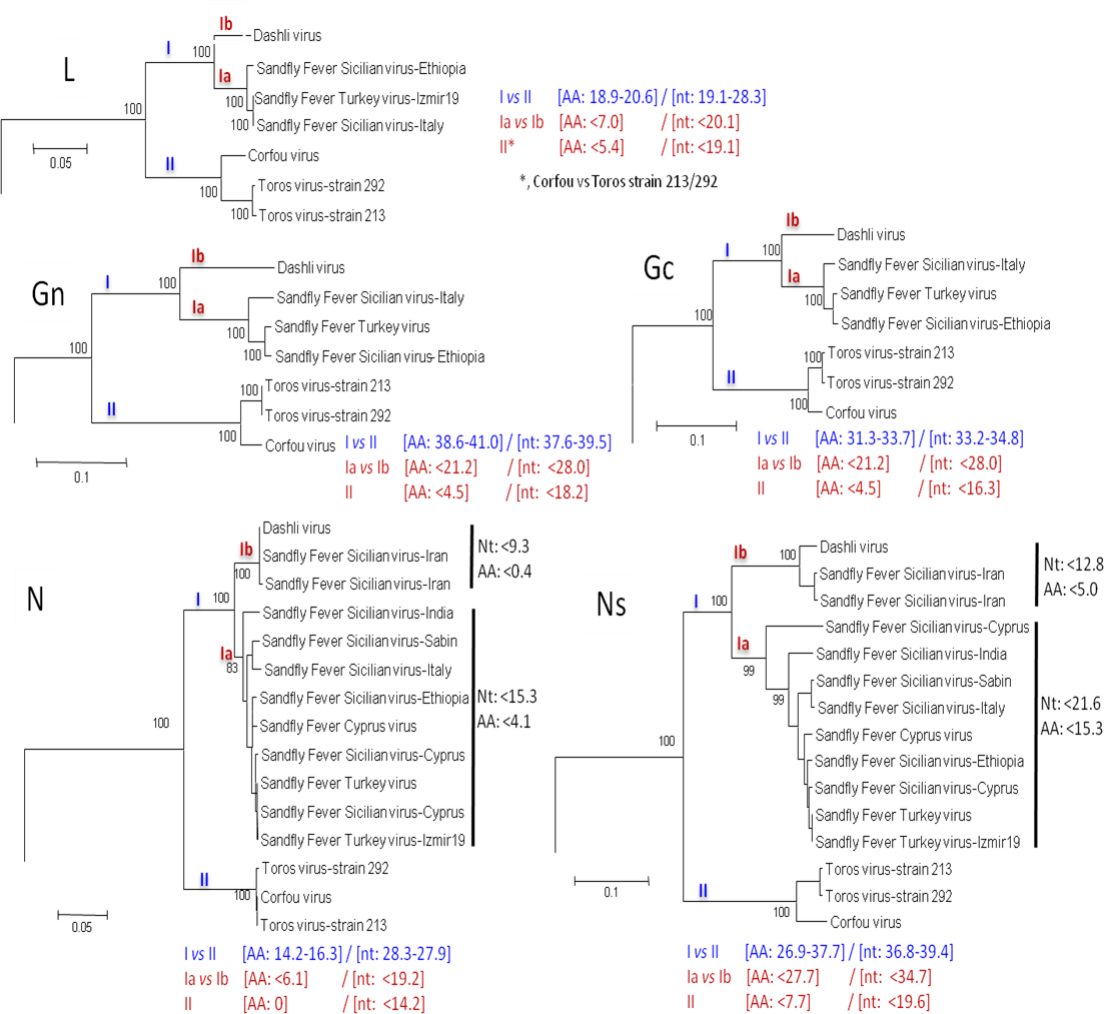
Phylogeny and proposed lineages and sublineages within the Sandfly fever Sicilian virus complex.

Regardless of the gene used, DASHV clustered with following viruses: SFSV from Iran, Italy, Ethiopia, SFCV, SFTV, CFUV and TORV, as supported by 100% bootstrap (Figs 2–7). Within this group, two lineages consisting of DASHV, SFSV strains, SFCV and SFTV on one hand (I), and of CFUV and TORV strains one the other hand (II) were also consistently observed and supported by 100% bootstrap values (Figs 2–7). Complete coding sequences of N and Ns genes were available for the aforementioned strains, but also for 6 additional strains consisting of 3 strains from Cyprus, 2 from Iran, 1 from India (Fig 5 & Fig 6); here, DASHV was consistently grouped with the two other Iranian strains (sublineage Ib) which are clearly distinct from other SFS-like viruses from Italy, Cyprus, Turkey, and Ethiopia (sublineage Ia).

### Phylogenetic analysis using partial polymerase sequences

Characteristics of these sequences are presented in S2 Table. The corresponding phylogenetic tree is presented in S2 Fig. As shown in the complete sequence phylograms, DASHV is clearly distinct from other viruses, which were split into 3 groups supported by bootstrap value >70%: (i) lineage I (99% bootstrap support) consisted of 11 sequences (lineage Ia) representing SFSV-Italy, SFSV-Ethiopia, SFSV-Tunisia, SFSV-Algeria, SFCV, and SFTV and of DASHV sequence (lineage Ib); (ii) lineage II was splitted into 3 sublineages, lineage IIa consisted of CFUV and the unique sequence of Chios-A virus (Greece) [32], lineage IIb consisted of 7 sequences of Utique virus [9], lineage IIc consisted of Toros virus together with two sequences of Girne 2 virus (Northern Cyprus) [32].

### Distribution of evolutionary distances upon pairwise comparison (Fig 8)

Regardless the protein used for analysis (L, N, Ns, Gn, Gc), distances observed between among DASHV, SFSV, TORV and CFUV were lower than the highest intraspecific distances. Of the 5 genes studied, L and N were the most suitable to determine cut-off values amenable to all phleboviruses.

**Fig 8:**
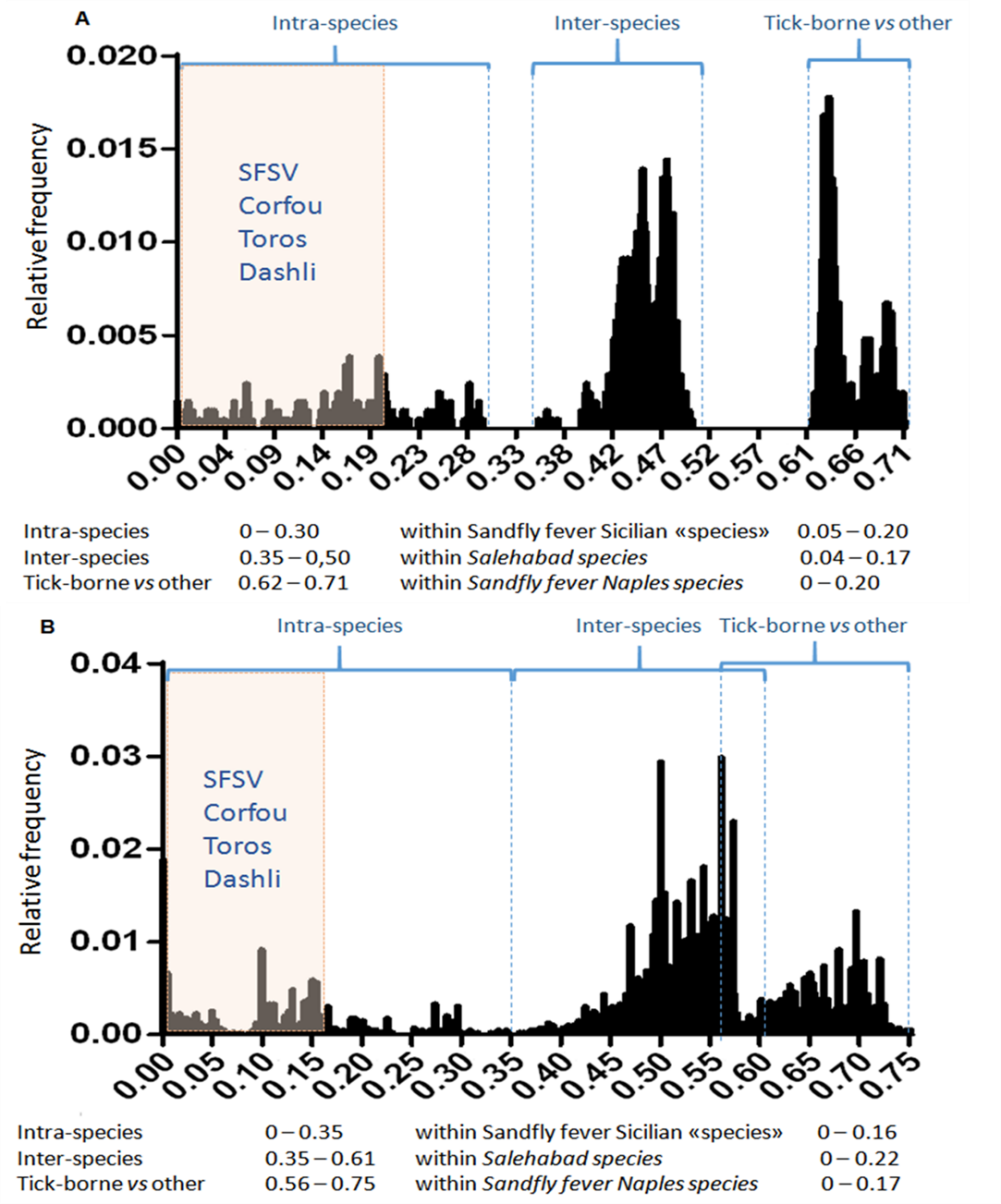
Distribution of evolutionary distances upon amino acid pairwise comparison of the complete open reading frame. The genetic distance is reported on the x-axis. Frequency of genetic distances is recorded on the y-axis. Distribution of the distances observed between sequences of the L (n = 46) and N (n = 62) ORFs. The shaded square represents AA distances observed between DASHV, SFSV, TORV and CFUV strains. Intra- and interspecific-ranges are indicated.

Using the complete polymerase gene AA sequences (Fig 8A), the cut-off value can be set at 0.35 for defining existing species: regarding SFSV, the highest genetic diversity is 0.206 supporting that DASHV, SFSV variants, Corfou and Toros viruses should be considered as members of the same species. The lowest genetic distance between these viruses and other phleboviruses are ≥0.435 (with mosquito- and sandfly-borne viruses) and ≥0.638 (with tick-borne phleboviruses)

Using the complete Nucleoprotein gene AA sequences (Fig 8B), the cut-off value can be set at 0.35 for defining existing species: regarding SFSV the highest genetic diversity is 0.16 supporting that DASHV, SFSV variants, Corfou and Toros viruses should be considered to belong to the same species. The lowest genetic distance between these viruses and other phleboviruses are ≥0.429 (with mosquito- and sandfly-borne viruses) and ≥0.616 (with tick-borne phleboviruses)

## Discussion

Despite the limited number of reported studies, Iran appears to be a rich source of phleboviruses. Indeed, prior to this study, four sandfly-borne phleboviruses had been isolated in Iran; SFSV, SALV, KARV, and THEV, each of which occupies a distinct genetic lineage [26, 28]. SFNV, the fifth sandfly-borne phlebovirus potentially present in Iran, was only indirectly identified through detection of neutralising antibodies in human and animal sera [27, 28]. The presence of neutralizing antibodies in human sera collected from 7 provinces over a wide geographical range demonstrated that SFSV, SFNV, and KARV virus were actively circulating in Iran before the 1970's [27]. There was no published data until now. Forty years after the seminal studies, we isolated DASHV from a pool of *Sergentomyia* spp and detected the same virus in a pool of *P*. *papatasi*, both collected in the Golestan province located in north-eastern Iran close to the Caspian Sea.

### Analysis of complete coding sequence

Genetic and phylogenetic analysis based on the complete coding sequence of the 5 viral genes together with homologous sequences of other phleboviruses indicates that DASHV belongs to a monophyletic group (100% bootstrap) consisting of several sandfly-borne phleboviruses: SFSV strains isolated in Italy and Ethiopia, SFTV (Turkey), SFCV (Cyprus), Corfou virus isolated in the eponymous Greek island, and Toros virus isolated in southern Anatolia (Turkey). Phylograms (Fig 7) show that DASHV is consistently more closely related with SFSV, SFTV and SFCV (lineage I) compared with Corfou and Toros viruses which form a distinct lineage (lineage II). The dichotomy of DASHV and SFSV (lineage I) *vs* Toros and CFU (lineage II) is consistently observed regardless the gene used for analysis (Fig 7).

In the S segment protein analysis DASHV grouped with two SFSV viruses isolated in Iran in 1975 from *Phlebotomus* spp. (Fig 5 & Fig 6, Genbank acc no EF201823 and EF201824) [33]. Currently, there are no sequences available for the L and M segment proteins of these viruses. The distances between DASHV and other lineage I sequences corresponding to viruses circulating outside of Iran ranged 4.5–19.8% and 18.3–29.6% for the AA and nt sequences of the L, M, and S proteins (S1 Table). In contrast, distances observed between DASHV and SFSV strains originating from Iran were lower than 0.0% and 9.3% for the N protein and 4.2% and 12.6% for the Ns protein, for AA and nt, respectively. Since the 3 Iranian strains are most closely related to each other than to any other phlebovirus, we propose to consider them as variant strains of DASHV which will be considered as a separate sublineage (Ib) within lineage I. Sublineage Ib is supported by 100% bootstrap values in N and Ns gene (sublineage Ib). Sublineage Ia includes all other SFSV and SFS-like viruses (SFTV, SFCV)(Figs 2–7). Obviously, during the past 40 years DASHV has evolved relatively slowly as showed by low sequence diversity (Fig 5 & Fig 6, S1 Table).

### Partial polymerase sequence analysis

Fig 8 correspond to the phylogenetic tree obtained with all publicly available sequences (partial and complete) of the polymerase gene; most of these sequences were obtained by using the RT-PCR assay designed by Sanchez-Seco et al. [29] (2003). The topology of the lineage I was unchanged although additional sequences obtained from Tunisia, and Algeria were included (S2 Fig). In contrast, 9 additional sequences clusterized within the lineage II which corresponded to Utique virus RNA (6 sequences; all detected in Tunisia) [34], Girne-2 virus RNA (2 sequences detected in north-eastern Turkey) [32], and Phlebovirus Chios-A RNA (1 sequence detected in the CSF of a Greek patient). When looking at Fig 8, it appears that lineage II may be divided into 3 sublineages although they are based on a 160-nt based analysis. Whether or not lineage II can be subdivided into at least 3 sublineages has to be confirmed when complete genomes will be sequenced.

### Taxonomic proposals

Although discovered seventy years ago, SFSV remains a tentative species within the genus *Phlebovirus*. Corfou virus, discovered in 1981, is in the same situation. Taking advantage of the recent increased number of complete sequences, we propose to consider that SFSV together with SFCV, SFTV and DASHV can represent a single species that could be named *Sandfly fever Sicilian virus* by analogy with the *Sandfly fever Naples* species. Whether CFUV and Toros viruses should be included in the Sandfly fever Sicilian virus species can be discussed; in our opinion, we would be inclined to merge all of these viruses in the same species, and to act as lumpers rather than splitters. However, it would be wise to wait for additional complete genome sequences of viruses included in the lineage II such as Chios, Utique and Girne 2 (S2 Fig).

### Entomological data

In Iran SFSV was isolated from *P*. *papatasi* and *Phlebotomus* spp. [28, 33]. In our study, the positive pools consisted of unengorged female *P*. *papatasi* and *Sergentomyia spp*. Sandfly-borne phleboviruses do not appear to possess a very restricted vector association: (i) SFTV and Corfou viruses were isolated from *P*. *major complex* sandflies in Turkey [35] and in Greece [24]; (ii) Sicilian-like virus sequences were detected in *P*. *ariasi* in Algeria [36] and also in *P*. *longicuspis*, *P*. *perniciosus* and *S*. *minuta* in Tunisia [9]. *Sergentomyia* spp. sandflies have long been considered unimportant vectors since they were not believed to feed on humans and mammals, but on reptiles. Recent results are increasingly questioning this point with cumulating evidence that *Sergentomyia spp*. could play a role in leishmaniasis in certain regions of the world [37–39]. Similarly for viruses, *Sergentomyia spp*. has been reported to be infected by a variety of different human pathogenic RNA viruses, such as Chandipura virus [40], Saboya virus [41], sandfly Sicilian-like virus [9], Toscana virus, Tete virus, and 2 unclassified viruses (ArD95737 and ArD 111740) [42]. It was recently demonstrated that some *Sergentomyia* species also feed on humans and/or mammals [43]. Indeed, there are recent direct studies which indicate that *Sergentomyia* species may be vectors of human and canine pathogens [37–39, 44, 45]. Whether future studies demonstrate that DASHV causes disease in humans, then further investigations will be necessary to identify unambiguously the vector.

**Fig 9:**
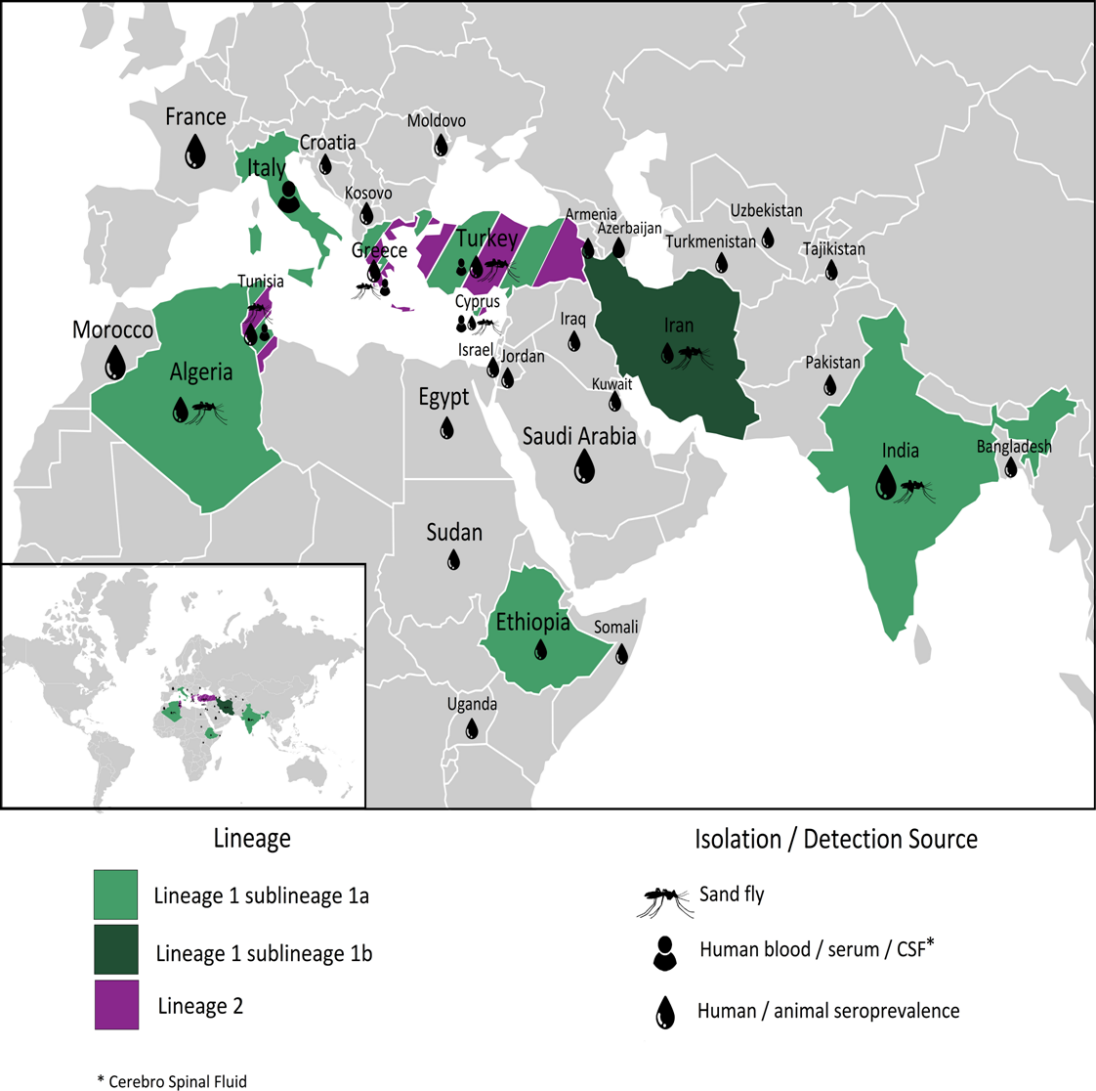
Geographic dispersal of Sandfly fever Sicilian virus.

The sandfly infection rate for DASHV (0.042%) is comparable with previous studies [8, 46–50]. However, since the trapping of sandflies in our study was performed in regions where the ecological and environmental conditions are very different, in Golestan the rate of infection in *Phlebotomus spp*. and *Sergentomyia spp*. is 1/904 (0.11%) and 1/1455 (0.068%), respectively; this suggests important circulation and possibly important exposure for human and non human vertebrates [34, 50]. This pleads for continuing investigation in this region through seroprevalence studies targeting vertebrates as recently in Europe and Africa. There are persistent evidence that SFSV and SFS-like viruses are occupying a large geographic area from South-western Europe to Middle east including northern Africa [51–53]. The majority of these viruses (Fig 9) cause epidemic or sporadic cases of infection in humans [22, 23, 25]. This should be incentive to perform studies in order to determine whether or not DASHV is involved in fever of unknown origin in the region. DASHV is genetically very close with SFSV. Although there is no data supporting that Corfou and Toros virus are human pathogens, there is considerable evidence of SFSV human cases of infection in Italy, Cyprus, Turkey, Ethiopia [18, 20, 23]. There is strong evidence that the different variants of SFSV cause febrile illness in humans either in sporadic or in epidemic cases [54]. Therefore, it is likely that DASHV can cause the same type of febrile illness. Seroprevalence studies have shown the presence of specific neutralising antibodies in humans in France, Italy, Cyprus and Israel [55–58]. It is therefore likely that Dashli virus can cause human infections and that these humans infection are probably febrile illness. Cohorts of patients presenting with unexplained fever in regions where sandfly vector are present should be tested for SFSV using PCR or serological techniques.

In Iraq, during an outbreak among US Army troops in 2007, 13 of 14 convalescent sera contained IgM antibodies specific for SFSV [59]. Specific IgG was also detected in Marine soldiers after self-reported febrile illness cases [60]. A variant strain of SFSV was also isolated from a human serum during an important outbreak in Ethiopia [23]. Accordingly, DASHV has the potential to cause human disease, which should be investigated through seroprevalence studies in Iran, specifically in Golestan. In addition, it would be worthwhile to develop a diagnostic test based on real-time RT-PCR to screen acute cases of febrile illness in the region where the virus has been isolated. The system that is described here was designed exclusively for DASHV. Because of several mismatches in the primers and probe sequences, it is likely to be of limited interest for the detection of the other viruses of the species. However, a real-time RT-PCR assay (detecting DASHV, SFSV, TORV and CFUV) has recently been described and could greatly help to better understand the medical impact of these viruses [51, 52].

In summary, a sandfly Sicilian-like phlebovirus was isolated from a non-engorged female pool of *Sergentomyia spp* and was detected in a non-engorged female pool of *P*. *papatasi* trapped in the Shordakesh in Dasliboroun village, in Iran. Its genetic characterization through complete sequencing of the three gene segments revealed that DASHV is closely related to other members of the Sandfly fever Sicilian virus species group. Further sandfly collections are required to strengthen our understanding of the circulation of DASHV and to update our knowledge of the distribution of sandfly-borne phleboviruses in Iran. Serological studies among animal and human populations are also required to investigate the pathogenicity of DASHV and its capacity to infect humans and other vertebrates since all the SFS-complex viruses with which it is closely related are pathogenic for humans.

## Materials and methods

### Sandfly trapping

Sandflies were trapped in several cities and villages from 10 provinces in Iran (Fig 1) using CDC Miniature Light Traps as previously reported [35] from June to September in 2009 and in 2011. Individual sandflies were identified using a stereomicroscope according to morphological characteristics [61, 62]. After identification, they were pooled based on species, sex, and location with up to 30 individuals per pool and placed in 1.5 ml tubes before storage at -80°C. When trapping was done in private areas, owners/residents were informed an gave oral permission for the study to be conducted on their land/in their residences.

### Virus detection

Pools of sandflies were homogenized in a final volume of 600μL as previously described [30] and 200-μL of the aliquot was used for viral nucleic acid (NA) extraction using the BioRobot EZ1-XL Advanced system (Virus Extraction Mini Kit, Qiagen). Five μL of NA was used for RT-PCR and nested-PCR assays with primers and protocols previously described [29, 48]. PCR products of the expected size were column-purified (Amicon Ultra Centrifugal filters, Millipore) and directly sequenced.

### Real-time RT-PCR for specific detection of DASHV

A real-time (rt) RT-PCR was designed in the nucleoprotein gene to detect specifically DASHV RNA. Sense (DASHV -N-FW [GATTGTAGAGGGCAGACCCG]) and reverse primers (DASHV -N-REV [TCCATTGCACTCCCAGGAAC]) were combined with the fluorogenic TaqMan probe (DASHV -N-Probe [6FAM-TGGACTGTCCAAGCTGTGGAGG-TAMRA]), and used with the Go Taq Probe 1-Step RT-qPCR (Promega) as previously reported [2].

### Virus isolation

Sandfly homogenates of 50μL were inoculated onto 12.5 cm^2^-tissue culture flasks containing Vero cell monolayers in EMEM, enriched with 1% Penicilin Streptomycin, 1% L-Glutamine 200 mM, 1% Kanamycin, and 3% Fungizone and 5mL of fresh EMEM containing 5% fetal bovine serum (FBS) was added after incubation at room temperature for 1 hr. To monitor the development of cytopathic effects the flasks were incubated at 37°C in 5% CO_2_ atmosphere and examined daily using an inverted microscope.

### Complete genome sequencing

Supernatant corresponding to passage 2 of DASHV infected Vero cells was used for complete genome characterization using Next Generation Sequencing (NGS) as previously described [2]; viral sequences were identified from the contigs based on the best BLAST similarity against reference databases using the CLC Genomics Workbench 7.0.4. Reads > 30 nucleotides long were trimmed using CLC Genomic Workbench 6.5, with a minimum of 99% quality per base and mapped to reference sequences on Genbank. Parameters were set such that each accepted read had to map to the reference sequence for at least 50% of its length, with a minimum of 80% identity to the reference. Sequence gaps were completed by PCR, designing specific primers based on NGS results and for the extremities using the primers previously defined [14], and PCR fragments were sequenced either by Sanger sequencing or by NGS. Once the complete genome was revealed, Sanger sequencing was performed through specific primers designed for the confirmation of the complete sequence.

### Pairwise genetic distances and phylogenetic analysis based on complete coding sequences

Complete coding regions of the S, M and L segments of phleboviruses were collected from Genbank (http://www.ncbi.nlm.nih.gov/genbank) and were aligned together with DASHV using the CLUSTAL algorithm of the MEGA 6 software [63]. Nucleotide (nt) and amino acid (AA) distances were calculated with the p-distance method. Neighbour-joining (NJ) analysis (Kimura 2-parameter model) was carried out using AA alignments, with 1000 bootstrap pseudoreplications. ML analysis was also performed using the best model defined for each gene. Since there was no obvious difference in the topology of the trees for viruses closely related with DASHV using either NJ or ML, final analysis including a larger set of sequences was performed using NJ.

### Phylogenetic analysis using partial polymerase sequences

A total of 24 homologous sequences most closely related with DASHV were aligned using Clustal W in MEGA6 [63]. The evolutionary history was inferred either by using the Maximum Likelihood method based on the Tamura 3-parameter model gamma distributed with invariant sites [63] or by the neighbour-joining method using the Kimura-2 parameter model. All positions with less than 95% site coverage were eliminated, so that they were a total of 160 positions in the final dataset. Evolutionary analysis was conducted in MEGA6.

### Distribution of evolutionary amino acid distances

Distribution of evolutionary distances upon amino acid pairwise comparison of the complete open reading frame were studied using complete coding sequences of the 5 ORFs. The genetic distance was reported on the x-axis. Frequency of genetic distances was recorded on the y-axis. Ranges were assessed for intraspecific and interspecific distances as previously described [64, 65].

### Accession numbers

The DASHV sequence has been deposited into the GenBank database with the corresponding accession numbers for L, M and S genomic segments KP771821, KP771822, and KP771823, respectively.

## Supporting information

S1 Fig CT values of the Real-time RT-PCR assay according to four-fold dilutions of the nucleic acid of the DASHV positive sandfly pools. X: CT values Y: serial four-fold dilutions; A: non-diluted, B: 1/4, C: 1/16, D: 1/64, E: 1/256, F: 1/1024, G: 1/4096. Blue line: Sample # 94 and red line: Sample # 131.
(TIF)

S2 Fig (TIF)

S1 Table Estimates (%) of evolutionary divergence between sequences of the polymerase (A), Gn glycoprotein (B), Gc glycoprotein (C), nucleocapsid (D), and non-structural (E) genes of the selected phleboviruses and the Dashli virus. The upper-right matrix represents pairwise distances between amino acids alignments. The lower-left matrix represents pairwise distances between nucleotides alignments.
(DOCX)

S2 Table Characteristics of sequences used in S2 Fig. (DOCX)
