# Supplementary figures and images for: Isolation and sequencing of Dashli virus, a novel Sicilian-like virus in sandflies from Iran; genetic and phylogenetic evidence for the creation of one novel species within the Phlebovirus genus in the Phenuiviridae family
Source: PLoS Negl Trop Dis. 2017 Dec 27;11(12):e0005978. doi: 10.1371/journal.pntd.0005978 (PMC5760094; doi:10.1371/journal.pntd.0005978)

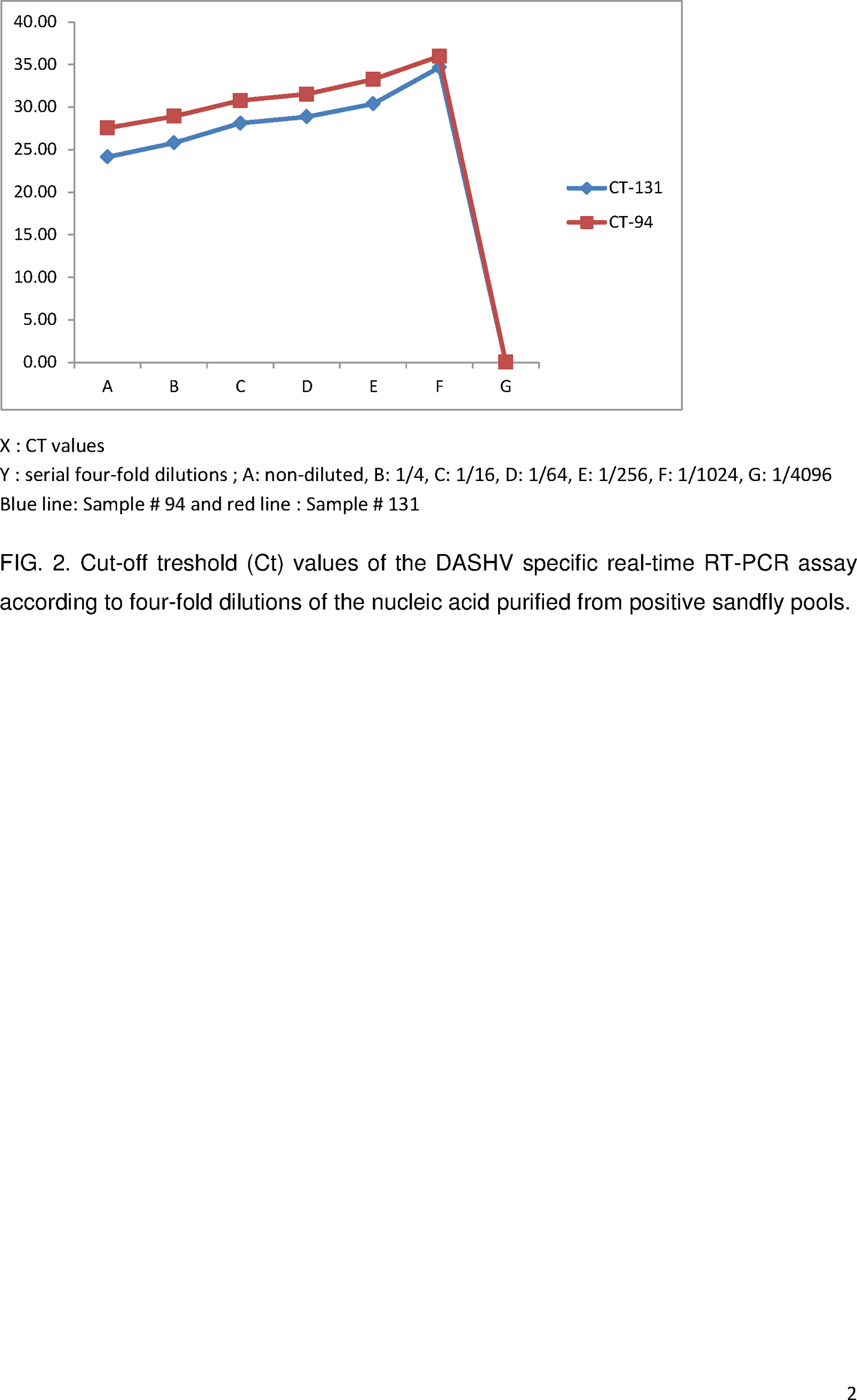

Supplement: S1 Fig — X: CT values Y: serial four-fold dilutions; A: non-diluted, B: 1/4, C: 1/16, D: 1/64, E: 1/256, F: 1/1024, G: 1/4096. Blue line: Sample # 94 and red line: Sample # 131. (TIF) [file pntd.0005978.s001.tif]

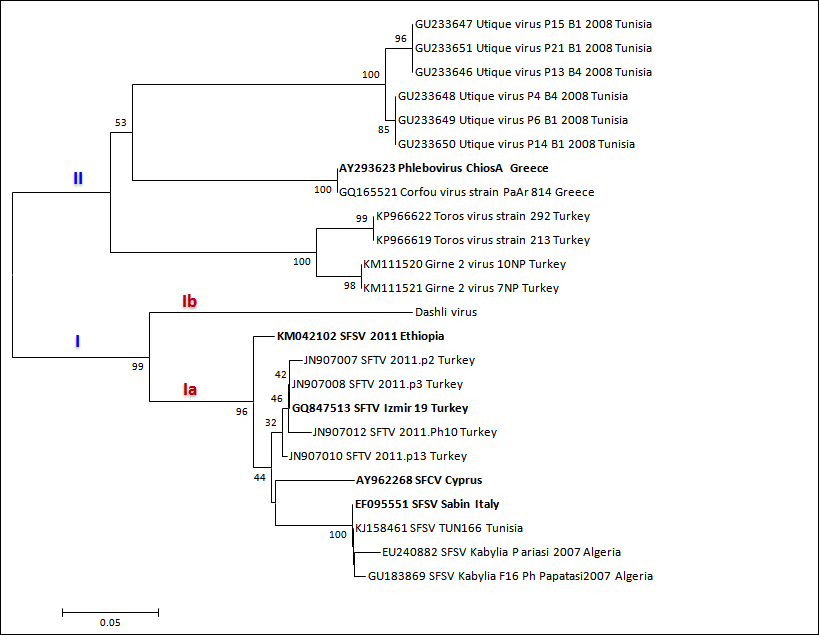

Supplement: S2 Fig — (TIF) [file pntd.0005978.s002.tif]
