# Supplementary material for: Isolation and sequencing of Dashli virus, a novel Sicilian-like virus in sandflies from Iran; genetic and phylogenetic evidence for the creation of one novel species within the Phlebovirus genus in the Phenuiviridae family
Source: PLoS Negl Trop Dis. 2017 Dec 27;11(12):e0005978. doi: 10.1371/journal.pntd.0005978 (PMC5760094; doi:10.1371/journal.pntd.0005978)
Supplement: S1 Table — Estimates (%) of evolutionary divergence between sequences of the polymerase (A), Gn glycoprotein (B), Gc glycoprotein (C), nucleocapsid (D), and non-structural (E) genes of the selected phleboviruses and the Dashli virus. The upper-right matrix represents pairwise distances between amino acids alignments. The lower-left matrix represents pairwise distances between nucleotides alignments. (DOCX) [file pntd.0005978.s003.docx]

A.

|  | L | **1**  **DASHV**  KP771819 | 2  SFSV  KM042102 | 3  SFTV  NC_015412 | 4  TORV  KP966619 | 5  CFUV  KR106179 | 6  KARV  KF297909 | 7  SALV  JX472403 | 8  SFNV  HM566172 | 9  THEV  JF939846 | 10  MASV  EU725771 | 11  TOSV  NC_006319 | 12  RVFV  DQ375430 |
| --- | --- | --- | --- | --- | --- | --- | --- | --- | --- | --- | --- | --- | --- |
| **1** | **DASHV** |  | **7.0** | **6.9** | **19.8** | **18.9** | **43.2** | **44.8** | **49.3** | **48.8** | **48.6** | **49.3** | **43.5** |
| 2 | SFSV | **20.0** |  | 1.1 | 20.6 | 19.5 | 43.7 | 44.9 | 49.5 | 49.1 | 48.9 | 49.2 | 43.6 |
| 3 | SFTV | **20.1** | 1.9 |  | 20.5 | 19.6 | 43.6 | 44.8 | 49.4 | 49.1 | 48.9 | 49.2 | 43.6 |
| 4 | TORV | **27.2** | 28.2 | 28.3 |  | 5.4 | 44.2 | 45.3 | 48.7 | 48.8 | 48.8 | 48.8 | 44.3 |
| 5 | CFUV | **28.1** | 19.1 | 27.8 | 19.1 |  | 43.2 | 44.0 | 47.7 | 47.8 | 48.0 | 47.8 | 42.9 |
| 6 | KARV | **41.0** | 41.0 | 40.9 | 41.3 | 40.5 |  | 42.0 | 46.7 | 46.6 | 46.2 | 47.3 | 40.7 |
| 7 | SALV | **41.9** | 41.9 | 42.1 | 41.3 | 41.4 | 40.3 |  | 44.9 | 44.7 | 45.1 | 45.0 | 40.4 |
| 8 | SFNV | **44.3** | 44.3 | 44.4 | 43.9 | 43.7 | 42.3 | 41.7 |  | 15.8 | 19.2 | 16.5 | 46.2 |
| 9 | THEV | **44.2** | 44.3 | 44.5 | 43.9 | 43.1 | 41.8 | 41.7 | 25.5 |  | 16.6 | 11.9 | 46.0 |
| 10 | MASV | **44.5** | 43.8 | 43.7 | 43.4 | 43.2 | 41.9 | 42.4 | 27.4 | 25.6 |  | 17.6 | 46.1 |
| 11 | TOSV | **44.3** | 43.9 | 43.9 | 43.9 | 44.0 | 43.0 | 42.1 | 26.4 | 23.0 | 27.0 |  | 46.4 |
| 12 | RVFV | **41.9** | 41.3 | 41.5 | 42.1 | 40.6 | 39.8 | 3.98 | 43.1 | 42.2 | 41.8 | 42.5 |  |

B.

|  | Gn | **1**  **DASHV**  KP771822 | 2  SFSV  U30500 | 3  SFSV  KM042103 | 4  SFTV  NC_015411 | 5  TORV  KP966620 | 6  CFUV  KR106178 | 7  KARV  KF297907 | 8  SALV  JX472404 | 9  SFNV  HM566171 | 10  THEV  JF939847 | 11  MASV  EU725772 | 12  TOSV  EU003177 | 13  RVFV  DQ380193 |
| --- | --- | --- | --- | --- | --- | --- | --- | --- | --- | --- | --- | --- | --- | --- |
| **1** | **DASHV** |  | **21.2** | **20.9** | **20.5** | **38.6** | **39.7** | **61.5** | **65.6** | **69.3** | **70.3** | **69.4** | **71.1** | **63.7** |
| 2 | SFSV | **28.0** |  | 5.3 | 4.5 | 40.7 | 41.0 | 61.3 | 67.4 | 72.2 | 74.0 | 73.0 | 72.6 | 66.3 |
| 3 | SFSV | **27.3** | 7.9 |  | 1.7 | 39.1 | 39.2 | 59.9 | 63.0 | 70.4 | 71.7 | 69.6 | 70.2 | 63.1 |
| 4 | SFTV | **27.4** | 7.4 | 2.0 |  | 39.4 | 39.5 | 59.9 | 63.4 | 70.2 | 71.7 | 69.3 | 69.8 | 63.3 |
| 5 | TORV | **37.6** | 38.2 | 37.7 | 37.6 |  | 4.9 | 55.3 | 61.8 | 65.2 | 65.8 | 66.4 | 67.1 | 59.4 |
| 6 | CFUV | **38.1** | 39.5 | 38.9 | 39.1 | 18.2 |  | 56.4 | 62.0 | 65.2 | 66.1 | 66.4 | 67.1 | 58.1 |
| 7 | KARV | **51.7** | 52 .0 | 50.8 | 50.5 | 46.6 | 47.9 |  | 61.1 | 68.1 | 69.9 | 68.5 | 69.4 | 63.3 |
| 8 | SALV | **54.5** | 55.6 | 53.1 | 53.5 | 52.9 | 52.2 | 50.4 |  | 65.4 | 68.1 | 65.7 | 69.4 | 65.4 |
| 9 | SFNV | **56.4** | 58.1 | 56.3 | 56.1 | 53.9 | 54.0 | 55.0 | 54.9 |  | 42.9 | 43.8 | 46.6 | 68.5 |
| 10 | THEV | **57.2** | 59.9 | 58.2 | 58.1 | 54.1 | 53.8 | 54.7 | 55.8 | 38.6 |  | 46.1 | 45.0 | 70.7 |
| 11 | MASV | **55.8** | 58.4 | 55.6 | 55.6 | 54.2 | 54.9 | 56.7 | 54.4 | 40.2 | 41.7 |  | 45.9 | 69.0 |
| 12 | TOSV | **57.2** | 58.5 | 56.1 | 56.3 | 54.3 | 55.4 | 56.3 | 58.3 | 42.0 | 40.8 | 43.0 |  | 68.9 |
| 13 | RVFV | **52.7** | 53.8 | 53.5 | 53.3 | 51.1 | 52.3 | 52.5 | 54.4 | 55.8 | 56.3 | 56.7 | 58.3 |  |

C.

|  | Gc | **1**  **DASHV**  KP771822 | 2  SFSV  U30500 | 3  SFSV  KM042103 | 4  SFTV  NC_015411 | 5  TORV  KP966620 | 6  CFUV  KR106178 | 7  KARV  KF297907 | 8  SALV  JX472404 | 9  SFNV  HM566171 | 10  THEV  JF939847 | 11  MASV  EU725772 | 12  TOSV  EU003177 | 13  RVFV  DQ380193 |
| --- | --- | --- | --- | --- | --- | --- | --- | --- | --- | --- | --- | --- | --- | --- |
| **1** | **DASHV** |  | **16.8** | **13.6** | **13.2** | **31.6** | **31.3** | **45.4** | **51.5** | **52.6** | **53.2** | **53.0** | **53.0** | **51.0** |
| 2 | SFSV | **25.6** |  | 4.7 | 3.9 | 32.5 | 31.3 | 48.3 | 55.9 | 55.6 | 55.7 | 56 .2 | 58 .1 | 51.3 |
| 3 | SFSV | **24.6** | 9.6 |  | 0.8 | 33.7 | 32.5 | 46.7 | 53.0 | 53.0 | 53.4 | 53.6 | 52.8 | 49.9 |
| 4 | SFTV | **24.3** | 8.4 | 1.8 |  | 33.3 | 32.1 | 46.7 | 53.2 | 53.0 | 53.4 | 53.8 | 52.8 | 50.1 |
| 5 | TORV | **33.2** | 34.3 | 34.1 | 34.8 |  | 4.5 | 47.6 | 53.6 | 56.8 | 56.5 | 56.0 | 54.1 | 53.3 |
| 6 | CFUV | **33.3** | 33.3 | 33.5 | 33.7 | 16.3 |  | 47.1 | 53.1 | 56.5 | 55.8 | 55.8 | 54.3 | 52.4 |
| 7 | KARV | **42.1** | 44.7 | 43.0 | 42.7 | 41.7 | 42.4 |  | 48.3 | 48.5 | 51.1 | 50.0 | 48.3 | 42.0 |
| 8 | SALV | **46.1** | 48.2 | 45.7 | 45.7 | 46.2 | 47.1 | 42.8 |  | 54.8 | 52.0 | 51.0 | 51.2 | 50.2 |
| 9 | SFNV | **45.2** | 49.7 | 46.0 | 46.2 | 46.9 | 48.2 | 44.5 | 46.1 |  | 24.5 | 28.5 | 27.2 | 51.3 |
| 10 | THEV | **45.7** | 50.6 | 46.5 | 46.8 | 47.7 | 48.1 | 45.7 | 47.2 | 28.6 |  | 25.3 | 26.4 | 53.0 |
| 11 | MASV | **45.2** | 49.0 | 45.6 | 45.7 | 46.3 | 46.5 | 44.2 | 47.1 | 31.9 | 31.3 |  | 22.3 | 52.3 |
| 12 | TOSV | **45.7** | 50.0 | 46.1 | 46.0 | 47.7 | 48.1 | 43.7 | 47.3 | 32.5 | 32.1 | 30.1 |  | 51.7 |
| 13 | RVFV | **45.3** | 47.7 | 45.4 | 45.6 | 46.9 | 48.0 | 40.1 | 45.7 | 45.6 | 48.7 | 46.0 | 48.2 |  |

D.

|  |  | **1**  **DASHV**  KP771823 | 2  SFSV  KM042104 | 3  SFSV  EF201823 | 4  SFSV  EF201824 | 5  SFSV  EF201827 | 6  SFSV  EF201826 | 7  SFSV  EF201825 | 8  SFSV  AJ811547 | 9  SFSV  EF201822 | 10  SFCV  GU119908 | 11  TORV  KP771821 | 12  CFUV  KR106179 | 13  SFTV  NC015413 | 14  KARV  KF297914 | 15  SALV  JX472405 | 16  SFNV  EF201829 | 17  THEV  JF939848 | 18  MASV  EU725773 | 19  TOSV  NC006318 | 20  RVFV  DQ380157 |
| --- | --- | --- | --- | --- | --- | --- | --- | --- | --- | --- | --- | --- | --- | --- | --- | --- | --- | --- | --- | --- | --- |
| **1** | **DASHV** |  | **4.5** | **0.0** | **0.4** | **4.9** | **4.9** | **5.3** | **5.7** | **4.5** | **4.9** | **15.0** | **15.0** | **4.9** | **47.3** | **55.1** | **56.5** | **55.3** | **57.7** | **56.9** | **46.9** |
| 2 | SFSV | **18.3** |  | 4.5 | 4.9 | 2.8 | 0.8 | 1.2 | 2.8 | 1.6 | 0.8 | 15.9 | 15.9 | 0.8 | 47.7 | 55.9 | 54.5 | 54.1 | 55.7 | 56.1 | 46.9 |
| 3 | SFSV | **9.3** | 18.7 |  | 0.4 | 4.9 | 4.9 | 5.3 | 5.7 | 4.5 | 4.9 | 15.0 | 15.0 | 4.9 | 47.3 | 47.3 | 55.1 | 55.3 | 57.7 | 56.9 | 46.9 |
| 4 | SFSV | **9.3** | 19.0 | 1.6 |  | 5.3 | 5.3 | 5.7 | 6.1 | 4.9 | 5.3 | 15.4 | 15.4 | 5.3 | 47.7 | 54.7 | 56.9 | 55.7 | 57.3 | 57.3 | 46.9 |
| 5 | SFSV | **18.7** | 15.3 | 18.2 | 18.3 |  | 3.3 | 3.7 | 4.1 | 2.8 | 2.4 | 14.2 | 14.2 | 3.3 | 47.3 | 56.3 | 54.9 | 54.5 | 55.7 | 56.1 | 47.8 |
| 6 | SFSV | **18.8** | 1.9 | 18.7 | 19.0 | 14.1 |  | 0.4 | 2.8 | 1.6 | 0.8 | 15.9 | 15.9 | 0.0 | 47.3 | 55.9 | 54.5 | 54.1 | 55.7 | 56.1 | 46.5 |
| 7 | SFSV | **18.8** | 2.0 | 19.0 | 19.2 | 14.2 | 0.4 |  | 3.3 | 2.0 | 1.2 | 16.3 | 16.3 | 0.4 | 47.7 | 55.9 | 54.5 | 54.1 | 55.7 | 56.1 | 46.5 |
| 8 | SFSV | **18.2** | 8.0 | 18.2 | 17.8 | 13.0 | 6.6 | 6.9 |  | 1.6 | 2.8 | 16.3 | 16.3 | 2.8 | 48.1 | 56.7 | 55.3 | 54.9 | 56.1 | 56.5 | 47.8 |
| 9 | SFSV | **17.8** | 7.5 | 17.8 | 17.3 | 12.6 | 6.1 | 6.4 | 0.5 |  | 1.6 | 15.4 | 15.4 | 1.6 | 47.7 | 56.3 | 55.3 | 54.9 | 56.1 | 56.5 | 47.3 |
| 10 | SFCV | **18.3** | 3.9 | 18.8 | 18.7 | 13.6 | 3.1 | 3.3 | 7.3 | 6.8 |  | 15.0 | 15.0 | 0.8 | 47.7 | 55.5 | 54.5 | 54.1 | 55.7 | 56.1 | 46.9 |
| 11 | TORV | **24 .0** | 24.9 | 25.1 | 25.2 | 25.6 | 25.2 | 25.2 | 25.7 | 25.6 | 25.1 |  | 0.0 | 15.9 | 45.6 | 52.7 | 58.0 | 57.1 | 59.6 | 58.0 | 46.9 |
| 12 | CFUV | **23.8** | 27.6 | 24.7 | 25.2 | 25 .7 | 27.8 | 27.9 | 27.0 | 26.8 | 27.5 | 14.2 |  | 15.9 | 45.6 | 52.7 | 57.3 | 56.5 | 58.9 | 57.3 | 46.9 |
| 13 | SFTV | **18.3** | 1.4 | 18.4 | 18.7 | 14.6 | 1.6 | 1.8 | 7.6 | 7.0 | 3.9 | 24.8 | 27.5 |  | 47.3 | 55.9 | 54.5 | 54.1 | 55.7 | 56.1 | 46.5 |
| 14 | KARV | **44.3** | 42.7 | 43.2 | 44.3 | 44.7 | 42.6 | 42.9 | 44.0 | 43.8 | 43.4 | 43.7 | 44.7 | 43.0 |  | 46.9 | 54.8 | 53.5 | 53.9 | 53.1 | 44.0 |
| 15 | SALV | **45.4** | 48.3 | 44.6 | 44.1 | 47.8 | 48.4 | 48.4 | 48.0 | 47.9 | 48.0 | 44.9 | 46.0 | 48.0 | 43.4 |  | 53.8 | 53.8 | 53.4 | 53.4 | 48.6 |
| 16 | SFNV | **49.6** | 48.4 | 48.2 | 48.6 | 47.7 | 48.4 | 48.2 | 48.9 | 48.6 | 48.8 | 49.0 | 48.8 | 48.4 | 47.4 | 44.1 |  | 11.9 | 13.0 | 9.9 | 49.8 |
| 17 | THEV | **47.2** | 48.2 | 47.6 | 47.6 | 47.6 | 48.1 | 48.1 | 48.6 | 48.4 | 48.6 | 48.0 | 48.6 | 47.8 | 47.3 | 45.3 | 21.1 |  | 16.5 | 15.4 | 50.6 |
| 18 | MASV | **46.5** | 48.1 | 47.3 | 47.4 | 48.0 | 48.1 | 48.0 | 48.0 | 47.7 | 48.0 | 48.8 | 48.5 | 47.7 | 47.9 | 44.1 | 22.4 | 20.7 |  | 14.2 | 49.8 |
| 19 | TOSV | **47.6** | 47.6 | 47.8 | 47.6 | 48.0 | 47.4 | 48.0 | 48.6 | 48.2 | 47.8 | 47.6 | 47.4 | 47.2 | 46.7 | 45.5 | 21.3 | 22.5 | 23.2 |  | 50.2 |
| 20 | RVFV | **41.1** | 40.8 | 40.1 | 40.3 | 40.7 | 40.8 | 47.3 | 39.3 | 39.0 | 40.8 | 42.0 | 41.5 | 40.7 | 43.0 | 42.4 | 45.7 | 44.6 | 43.9 | 46.1 |  |

E.

|  |  | **1**  **DASHV**  KP771823 | 2  SFSV  KM042104 | 3  SFSV  EF201823 | 4  SFSV  EF201824 | 5  SFSV  EF201827 | 6  SFSV  EF201826 | 7  SFSV  EF201825 | 8  SFSV  AJ811547 | 9  SFSV  EF201822 | 10  SFCV  GU119908 | 11  TORV  KP771821 | 12  CFUV  KR106179 | 13  SFTV  NC015413 | 14  KARV  KF297914 | 15  SALV  JX472405 | 16  SFNV  EF201829 | 17  THEV  JF939848 | 18  MASV  EU725773 | 19  TOSV  NC006318 | 20  RVFV  DQ380157 |
| --- | --- | --- | --- | --- | --- | --- | --- | --- | --- | --- | --- | --- | --- | --- | --- | --- | --- | --- | --- | --- | --- |
| **1** | **DASHV** |  | **19.8** | **4.2** | **5.0** | **17.9** | **26.4** | **19.1** | **21.3** | **21.0** | **19.8** | **36.2** | **26.9** | **19.1** | **73.5** | **76.9** | **83.1** | **90.4** | **88.0** | **86.7** | **76.0** |
| 2 | SFSV | **29.6** |  | 20.2 | 20.6 | 4.8 | 12.0 | 1.2 | 4.4 | 3.6 | 2.0 | 35.1 | 37.1 | 1.2 | 70.2 | 76.1 | 81.4 | 88.0 | 84.7 | 84.8 | 73.2 |
| 3 | SFSV | **12.6** | 27.7 |  | 0.8 | 18.8 | 27.3 | 19.5 | 21.8 | 21.5 | 20.3 | 36.9 | 37.7 | 19.2 | 73.5 | 77.7 | 82.5 | 89.6 | 88.7 | 86.6 | 76.4 |
| 4 | SFSV | **12.8** | 27.4 | 2.0 |  | 18.8 | 27.7 | 19.9 | 22.2 | 21.8 | 20.7 | 36.9 | 37.7 | 19.5 | 73.9 | 78.1 | 82.5 | 89.6 | 88.7 | 86.6 | 76.0 |
| 5 | SFSV | **27.1** | 15.2 | 25.5 | 25.3 |  | 15.3 | 5.7 | 7.6 | 7.3 | 6.5 | 34.6 | 37.3 | 5 .7 | 70.8 | 76.2 | 83.5 | 88.9 | 86.4 | 85.4 | 72.8 |
| 6 | SFSV | **34.7** | 11.0 | 32.9 | 32.9 | 21.6 |  | 10.7 | 14.2 | 13.4 | 12.3 | 37.8 | 39.8 | 10.7 | 69.6 | 78.4 | 81.3 | 88.8 | 85.6 | 84.2 | 75.5 |
| 7 | SFSV | **29.4** | 2.5 | 27.5 | 27.3 | 15.1 | 8.7 |  | 4.6 | 3.8 | 1.9 | 35.0 | 36.9 | 0.8 | 69.6 | 75.8 | 82.2 | 88.9 | 85.3 | 85.4 | 73.2 |
| 8 | SFSV | **27.6** | 9.0 | 27.6 | 27.7 | 16.3 | 15.3 | 7.8 |  | 0.8 | 4.6 | 36.9 | 38.5 | 4.5 | 70.4 | 75.4 | 83.1 | 88.2 | 84.9 | 85.1 | 73.6 |
| 9 | SFSV | **27.9** | 8.7 | 27.6 | 27.7 | 16.0 | 15.1 | 7.5 | 0.3 |  | 3.8 | 36.2 | 37.7 | 3.8 | 70.4 | 75.4 | 82.6 | 88.1 | 84.5 | 85 .4 | 73.6 |
| 10 | SFCV | **29.4** | 4.2 | 28.5 | 28.1 | 16.3 | 11.4 | 3.1 | 7.3 | 7.0 |  | 35.4 | 36.9 | 2.7 | 69.6 | 75.0 | 81.7 | 88.1 | 84.9 | 85.8 | 73.6 |
| 11 | TORV | **36.8** | 36.3 | 37.1 | 37.4 | 36.4 | 39.5 | 35.9 | 36.9 | 36.8 | 36.9 |  | 7.7 | 35.0 | 36.2 | 77.8 | 82.5 | 87.9 | 83.1 | 84.7 | 76.0 |
| 12 | CFUV | **39.2** | 38.9 | 38.8 | 39.4 | 37.2 | 41.8 | 38.1 | 37.8 | 37.7 | 38.5 | 19.6 |  | 36.5 | 73.4 | 76.4 | 82.5 | 89.5 | 85.9 | 86.5 | 76.7 |
| 13 | SFTV | **28.8** | 2.6 | 26.9 | 26.7 | 14.9 | 10.0 | 2.3 | 8.4 | 8 .3 | 3.8 | 36.4 | 37.8 |  | 69.6 | 75.8 | 82.3 | 89.0 | 85.3 | 85.5 | 73.2 |
| 14 | KARV | **61.1** | 56.4 | 59.5 | 59.7 | 58.1 | 57.5 | 56.2 | 56.7 | 56.8 | 56.3 | 60.4 | 59.1 | 55.6 |  | 70.5 | 80.4 | 88.6 | 88.1 | 86.7 | 71.4 |
| 15 | SALV | **64.0** | 62.4 | 64.0 | 63.3 | 62.6 | 63.7 | 61.5 | 62.1 | 62.1 | 61.3 | 62.9 | 61.9 | 61.8 | 59.4 |  | 83.4 | 87.1 | 82.9 | 85.6 | 81.4 |
| 16 | SFNV | **66.4** | 62.2 | 66.1 | 66.1 | 63.0 | 64.1 | 63.5 | 63.1 | 62.8 | 63.2 | 64.4 | 64.3 | 63.2 | 68.0 | 67.2 |  | 56.7 | 58.0 | 51.0 | 82.2 |
| 17 | THEV | **67.2** | 64.7 | 65.7 | 66.4 | 66.4 | 64.9 | 66.4 | 64.5 | 64.7 | 66 .4 | 65.3 | 67.9 | 65.9 | 67.4 | 70.0 | 50.4 |  | 56.3 | 43.7 | 86.9 |
| 18 | MASV | **65.1** | 65.7 | 66.1 | 66.7 | 65.6 | 65.9 | 66.3 | 66.3 | 66.1 | 66.5 | 62.3 | 65.5 | 66.0 | 67.2 | 65.4 | 50.5 | 49.8 |  | 54.8 | 87.2 |
| 19 | TOSV | **67.2** | 65.3 | 65.5 | 65.7 | 66.9 | 64.4 | 65.7 | 65.0 | 65.1 | 66.3 | 68.5 | 64.7 | 65.8 | 66.4 | 67.7 | 46.6 | 40.6 | 47.5 |  | 86.5 |
| 20 |  |  |  |  |  |  |  |  |  |  |  |  |  |  |  |  |  |  |  |  |  |
