# Supplementary material for: Isolation and sequencing of Dashli virus, a novel Sicilian-like virus in sandflies from Iran; genetic and phylogenetic evidence for the creation of one novel species within the Phlebovirus genus in the Phenuiviridae family
Source: PLoS Negl Trop Dis. 2017 Dec 27;11(12):e0005978. doi: 10.1371/journal.pntd.0005978 (PMC5760094; doi:10.1371/journal.pntd.0005978)
Supplement: S2 Table — (DOCX) [file pntd.0005978.s004.docx]

**S2 Table. Characteristics of sequences used in S2 Figure**

| location | date | virus | Detected or isolated in | Genbank  acc no |
| --- | --- | --- | --- | --- |
| Northern Cyprus | July 2013 | Girne 2 10NP | *P perfiliewi*  (Pool, 20 females) | KM111520 |
| Northern Cyprus | July 2013 | Girne 2 7NP | *P perfiliewi*  (Pool, 20 females) | KM111521 |
| Turkey, Southern Anatolia | 2012 | Toros 213 | *Phlebotomus spp*  (Pool, 20 males) | KP966619 |
| Turkey, Southern Anatolia | 2012 | Toros 292 | *Phlebotomus spp*  (Pool, 20 males) | KP966622 |
| Greece, Chios island |  | Chios-A | Human, CSF | AY293623 |
| Tunisia | 2008 | Utique P4_B4 | *P perniciosus* | GU233648 |
| Tunisia | 2008 | Utique P6_B1_2008 | *P perniciosus* | GU233649 |
| Tunisia | 2008 | Utique P23_B3_2008 | *P perniciosus* | GU233652 |
| Tunisia | 2008 | Utique P14_B1_2008 | *P longicuspis* | GU233650 |
| Tunisia | 2008 | Utique P13_B4_2008 | *P perniciosus* | GU233646 |
| Tunisia | 2008 | Utique P21_B1_2008 | *Sergentomyia minuta* | GU233651 |
| Tunisia | 2008 | Utique P15_B1_2008 | *P perniciosus* | GU233647 |
| Greece, Corfou island | 1981 | Corfou Pa Ar 814 | *P major* | GQ165521 |
| Cyprus | 2002 | Sandfly fever Sicilian Cyprus | Human, blood | AY962268 |
| Turkey, Izmir | 2008 | SFTV Izmir 19 | Human, serum | GQ847513 |
| Turkey | 2011 | SFTV2011.p3 | Human, serum | JN907008 |
| Turkey | 2011 | SFTV2011.p2 | Human, serum | JN907007 |
| Turkey | 2011 | SFTV2011.Ph10 | *Phlebotomus spp* | JN907012 |
| Turkey | 2011 | SFTV2011.p13 | Human, serum | JN907010 |
| Ethiopia | 2011 | SFSV Ethiopia2011 | Human, serum | KM042102 |
| Tunisia | 2011 | SFSV 166 | Human, CSF | KJ158461 |
| Italy | 1943 | SFSV Sabin | Human, serum | EF095551 |
| Italy | - | SFSV Italy | Human, serum | EU240882 |
| Algeria, Kabylia | 2007 | SFSV Kabylia F16 | *Ph ariasi* | GU183869 |
| Iran, Golestan | 2011 | Dashli | *P papatasi / Sergentomyia spp* | KP771821 |

CSF, Cerebro-Spinal Fluid
